# Lgl1 controls NG2 endocytic pathway to regulate oligodendrocyte differentiation and asymmetric cell division and gliomagenesis

**DOI:** 10.1038/s41467-018-05099-3

**Published:** 2018-08-21

**Authors:** Mathieu Daynac, Malek Chouchane, Hannah Y. Collins, Nicole E. Murphy, Noemi Andor, Jianqin Niu, Stephen P. J. Fancy, William B. Stallcup, Claudia K. Petritsch

**Affiliations:** 1 Department of Neurological Surgery, University of California San Francisco, San Francisco, CA 94143 USA; 2 Department of Pediatrics, University of California San Francisco, San Francisco, CA 94158 USA; 3 Department of Neurology, University of California San Francisco, San Francisco, CA 94158 USA; 4 Sanford Burnham Prebys Medical Discovery Institute, San Diego, CA 92037 USA; 5 Brain Tumor Center, University of California San Francisco, San Francisco, CA 94158 USA; 6 Helen Diller Comprehensive Cancer Center, University of California San Francisco, San Francisco, CA 94158 USA; 7 Eli and Edythe Broad Center of Regeneration Medicine and Stem Cell Research, University of California San Francisco, San Francisco, CA 94143 USA; 8 Present Address: Department of Neurobiology, Stanford University, Stanford, CA 94305 USA; 9 Present Address: Department of Medicine, Stanford University, Stanford, CA 94305 USA

## Abstract

Oligodendrocyte progenitor cells (OPC) undergo asymmetric cell division (ACD) to generate one OPC and one differentiating oligodendrocyte (OL) progeny. Loss of pro-mitotic proteoglycan and OPC marker NG2 in the OL progeny is the earliest immunophenotypic change of unknown mechanism that indicates differentiation commitment. Here, we report that expression of the mouse homolog of *Drosophila* tumor suppressor Lethal giant larvae 1 (Lgl1) is induced during OL differentiation. *Lgl1* conditional knockout OPC progeny retain NG2 and show reduced OL differentiation, while undergoing more symmetric self-renewing divisions at the expense of asymmetric divisions. Moreover, *Lgl1* and hemizygous *Ink4a/Arf* knockouts in OPC synergistically induce gliomagenesis. Time lapse and total internal reflection microscopy reveals a critical role for Lgl1 in NG2 endocytic routing and links aberrant NG2 recycling to failed differentiation. These data establish Lgl1 as a suppressor of gliomagenesis and positive regulator of asymmetric division and differentiation in the healthy and demyelinated murine brain.

## Abstract (web summary)

Oligodendrocyte progenitor cells (OPCs) undergo asymmetric cell division, and disruption of such mechanism can generate oligodendroglioma precursors. Here, Daynac and colleagues show that Lgl1 regulates asymmetric division and differentiation of OPCs by interfering with the endocytosis pathway, and that Lgl1 knockout can lead to gliomagenesis.

## Introduction

Adult oligodendrocyte progenitor cells (OPC) expressing the proteoglycan NG2 (CSPG4) continuously divide and generate differentiating oligodendrocytes (OL) throughout adulthood^1^. OPC divisions are frequently asymmetric and generate progeny of distinct fate, where only one of the daughter cells preserves NG2 expression and the opposite daughter cell downregulates NG2^2,3^. Thus far, asymmetric distribution of NG2 protein is the earliest immunophenotypic change amongst daughter cell pairs generated by asymmetric cell division (ACD). NG2-positive daughter cells proliferate at higher rates than NG2-negative cells, showing that the early phenotypic asymmetry correlates with distinct short-term fate^2^. NG2 contributes to establishing this cell fate bias within newly generated cell pairs after ACD. NG2 binds platelet-derived growth factor-AA (PDGF-AA) and PDGF receptor alpha (PDGFRα) and thereby enhances PDGFRα signaling and promotes timely OPC proliferation^4–7^. Moreover, long-term fate tracking of the ACD progeny showed that early NG2 asymmetry permanently affects cell fate. The NG2-positive progeny of ACD retain OPC characteristics while the NG2-negative progeny upregulate CC1, a marker for commitment to the OL fate, and eventually differentiate^2,8,9^. Importantly, chemical-induced demyelination increases the rates of NG2 asymmetry^9^. Collectively, the data underline that asymmetric distribution of NG2 marks and also actively generates different OPC progeny. Furthermore, ACD balances OPC proliferation with differentiation in the normal brain and generates OL in demyelinated lesions to contribute to remyelination^2,3,9,10^. It is unclear how the downregulation of NG2 protein is achieved in the differentiating oligodendrocyte. This lack of mechanistic insights into ACD and early differentiation limits our understanding of brain homeostasis.

OPC give rise to glioma in genetically engineered mouse models^11–14^. When undergoing neoplastic transformation, OPC show higher rates of symmetric self-renewing divisions at the expense of ACD^2^. These data suggest that downregulation of NG2 in OPC progeny is critical for ACD, differentiation, and attenuation of tumorigenesis^2^.

Surface levels of the membrane-spanning NG2 protein are regulated by clathrin-mediated endocytosis in mouse embryonic fibroblasts^15^. Clathrin-mediated or receptor-mediated endocytosis is a multi-step process, whereby membrane-localized proteins are engulfed as cargo in clathrin-coated pits, which then bud off the membrane to form the early endosome. Cargo proteins are then sorted into either the recycling endosome and re-integrated into the membrane or targeted to the late endosome and subsequently the lysosome for degradation^16^. It is not known whether NG2 trafficking by the endocytic pathway is important for NG2 downregulation and OL differentiation.

The WD40 repeat-containing protein lethal giant larvae (Lgl) was initially characterized as a tumor suppressor gene, in *Drosophila melanogaster*^17^, and it was subsequently shown to mediate ACD of *Drosophila* neuroblasts^18^. Lgl is an evolutionary conserved protein that initiates cell polarity by recruiting proteins to membrane subdomains (for review, see ref. ^19^). As one of two mammalian *Lgl* genes^20^, *Lgl1* is highly expressed in the brain^21^. *Lgl1* knockout studies during mouse embryogenesis have revealed a function for Lgl1 in polarity and adherens junction integrity in neuroepithelial cells^22^, and in suppressing proliferation of dorsal telencephalon radial glial progenitors at early postnatal stages^23^. A bona-fide tumor suppressor role for Lgl1 in gliomagenesis is supported by studies showing that loss of tumor suppressor *PTEN*, a frequent and early driver mutation in glioblastoma^24^, inhibits Lgl1 activity^25^. Constitutively active Lgl1 inhibits invasion and induces differentiation of primary human glioblastoma cells^26^. Nevertheless, a tumor suppressive role for Lgl1 in OPC has not been investigated yet.

Here, we report that *Lgl1* expression is upregulated during OL differentiation and that Lgl1 protein is detected in committed OL in the adult murine brain. *Lgl1* conditional knockout (cKO) OPC show a differentiation defect characterized by an aberrant co-expression of NG2 with OL commitment markers. Moreover, in *Lgl1* cKO OPC rates of ACD are reduced while rates of symmetric, self-renewing divisions and proliferation are increased, in both intact and chemically demyelinated corpus callosum (CC). *Lgl1* knockout synergizes with hemizygous *Ink4a/Arf* knockout in OPC to induce gliomagenesis. Time lapse imaging of surface-labeled, endocytosed NG2 shows decreased co-localization with the lysosome in *Lgl1* cKO OPC. Additionally, total internal reflection microscopy reveals that in *Lgl1* cKO OPC NG2 bypasses degradation and is rather recycled to the membrane. Aberrant NG2 recycling is linked to defective OPC differentiation. Thus, we establish Lgl1 as a positive regulator of differentiation and ACD and suppressor of gliomagenesis. We suggest that Lgl1 promotes differentiation by supporting NG2 routing to the lysosome.

## Results

### *Lgl1* expression is upregulated in OL differentiation

**Fig. 1:**
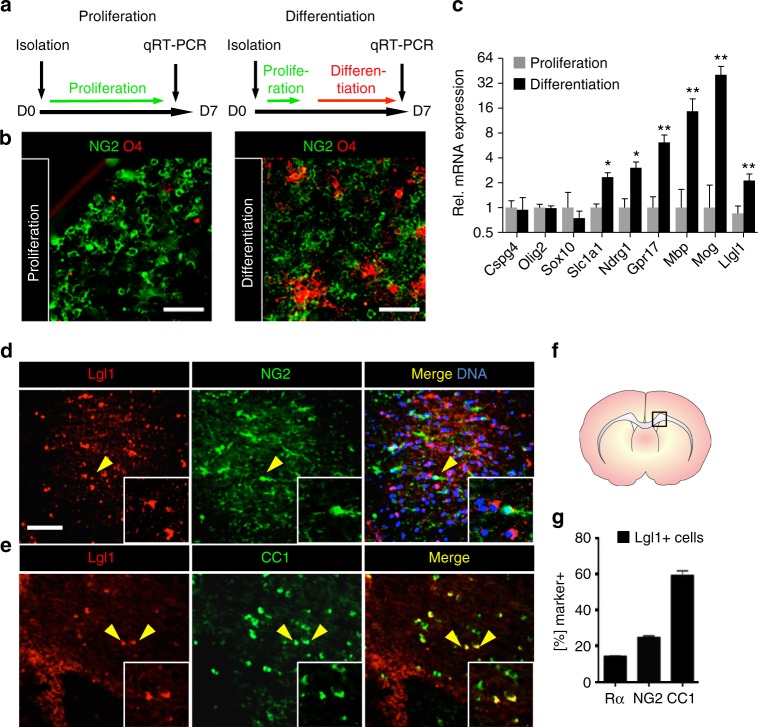
*Lgl1* is expressed in oligodendrocyte progenitor cells (OPC) and its expression increases with differentiation into oligodendrocytes. **a** Scheme of OPC treatment before qRT-PCR. **b** Immunofluorescence for OPC marker NG2 and oligodendrocyte differentiation marker O4 of corpus callosum-derived OPC under proliferating or differentiating conditions. Differentiation increases O4-positive cell frequency. Scale bar = 100 µm. **c** Graph representing mRNA expression level changes with differentiation. qRT-PCR results are displayed on a log_2_ scale and normalized to proliferative conditions. Data represented as mean ± s.e.m. of five independent experiments (*n* = 5, *p < 0.05, **p < 0.01, Mann–Whitney test). **d**, **e** Co-immunostaining of P35 wildtype mouse corpus callosum for Lgl1 and OPC marker NG2 (top panels) or differentiation marker CC1 (bottom panels). Insets are high magnification panels of Lgl1 positive cells (yellow arrows). Scale bar: 100 μm. **f** Schematic of coronal section of a P60 mouse showing the corpus callosum where cells were counted. **g** Quantification of PDGFRα-positive, NG2-positive, and CC1-positive cells per total Lgl1-positive cells. 14% were PDGFRα-positive, 22% were NG2-positive, and 59% were CC1-positive. Data are represented as mean ± s.e.m. of four independent experiments

To unravel the mechanism for NG2 down-regulation, we searched the RNA-Seq transcriptome database^27^ for conserved ACD regulator genes with altered expression in OL versus OPC. We noticed a four-fold increase of *Lgl1* (*Llgl1*) transcript in newly formed OL versus OPC, suggesting that *Llgl1* expression is increased upon OL differentiation. To directly test this notion, OPC were isolated from the white matter area of the CC of postnatal days 30–60 (P30–P60) mice and cultured in parallel under proliferation and differentiation conditions (Fig. 1a). We performed immunofluorescence staining for OPC marker NG2 and mature OL marker O4 to show differentiation. As expected, we found O4-expressing cells displaying a branched OL morphology only under differentiation but not under proliferation conditions (Fig. 1b). We used quantitative real-time PCR (qrt-PCR) to assess changes in *Llgl1* transcript levels with differentiation and aging. Analyses reveal a significant increase in *Llgl1* transcript under differentiation conditions (2.5-fold, *p* < 0.01) (Fig. 1c), along with differentiation markers *Mbp*, *Slc1a1*, *Ndrg1*, *GPR17*, and *Mog*. We noted that OPC markers, such as *Cspg4 (NG2), Olig2*, and *Sox10* are unchanged. *Llgl1* transcript levels increase with age, being higher at P60 in OPC, and reaching a peak at P180 in OL (Supplementary Figs. 1d, e).

To test whether Lgl1 protein expression is detectable in differentiated OL in situ, as suggested by our transcript analyses, we co-immunostained P35 wildtype mouse brain sections for Lgl1, OPC markers PDGFRα, and NG2 (Fig. 1d, Supplementary Fig. 1a), and committed OL marker CC1 (Fig. 1e)^8^. A quantification of all detectable Lgl1-positive cells in the CC revealed that more Lgl1-expressing cells are CC1 positive (59%) than PDGFRα (14%) and NG2 (22%) positive, respectively (Fig. 1f, g). Expression of Lgl1 is undetectable in NG2-positive OPC in the subventricular zone (Supplementary Figs. 1b, c).

Hence, Lgl1 expression increases with differentiation and overlaps largely with differentiating OL, suggesting therefore its potential role in a differentiation mechanism.

### *Lgl1* knockout in OPC blocks OL differentiation

**Fig. 2:**
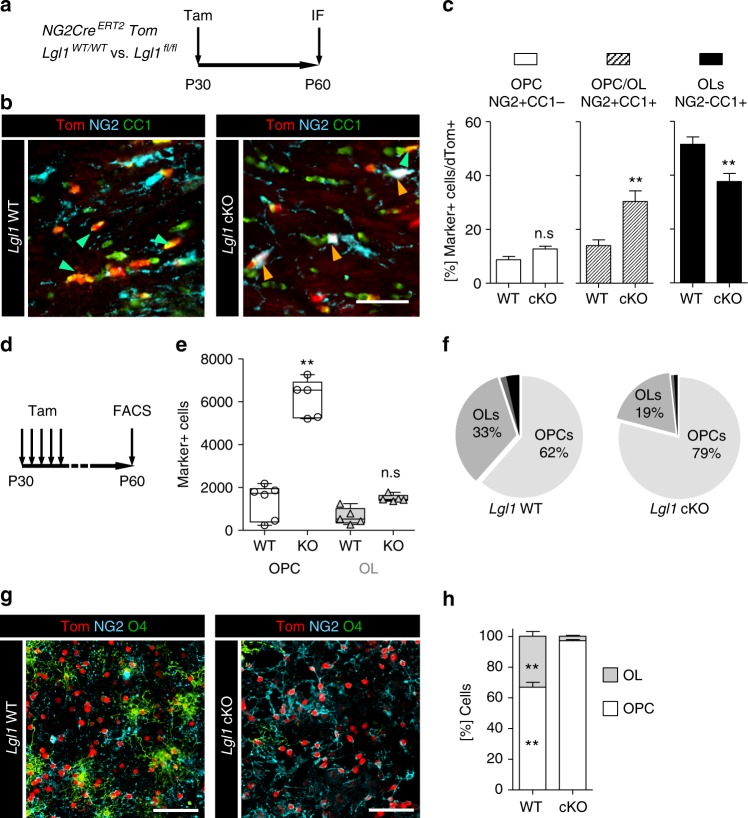
*Lgl1* knockout blocks OPC to OL differentiation. **a** Schematic of approach for clonal labeling and NG2 Cre-conditional deletion of *Lgl1* in OPC. **b** Representative triple immunostaining of corpus callosum of *Lgl1* WT and *Lgl1* cKO from mice treated as in (**a**) for red fluorescent protein (Tom), OPC marker NG2 and differentiation marker CC1. Green arrows point to Tom+CC1+ oligodendrocytes. Orange arrows in the *Lgl1* cKO panel point to Tom+NG2+CC1+ cells. Scale bar: 50 µm. **c** Quantification of NG2+, CC1+, and NG2+CC1+ cells ratios amongst Tom+ cells. Data are represented as mean ± s.e.m. from the analyses of 4 Lgl1 WT vs. 4 Lgl1 cKO treated mice (**p < 0.01, Mann–Whitney test). **d** Schematic of broad deletion of Lgl1 in P30 mice followed by FACS at P60. **e** Plot showing quantification of OPC (white) and OL (gray) in mice treated following scheme in (**d**). Data are represented as box & whiskers ± s.e.m. from the analyses of 6 Lgl1 WT vs. 5 *Lgl1* cKO treated mice (**p < 0.01, Mann–Whitney test). **f** Pie charts of quantification for NG2+, O4+, astrocyte marker GLAST+ and neuroblast marker CD24+ cells amongst Tom+ cells, after Lgl1 knockout at P60 (see also Supplementary Fig. 2b). Data are represented as percentages from the analyses of 6 *Lgl1* WT vs. *5 Lgl1* cKO mice (OPC *p* < 0.01; OL *p* < 0.01). **g** Representative immunostaining for NG2 and O4 of OPC and OL isolated from *GFAP-Cre, Tom*, *Lgl1*^*wt/wt*^ and, *GFAP-Cre*^*ERT2*^, *Tom*, *Lgl1*^*fl/fl*^ mice. Scale bars: 100 µm. **h** Quantification of NG2 and O4 cells amongst all red fluorescent protein (Tom) positive cells show that *Lgl1* knockout strongly blocks the differentiation of OPC into O4+OL. Data are represented as mean ± s.e.m. from the independent analyses of 4 *Lgl1* WT vs. 4 *Lgl1* cKO mice (**p < 0.01, Mann–Whitney test)

The expression pattern suggests that Lgl1 positively regulates OL differentiation. In order to investigate a putative role for Lgl1 in OPC differentiation, we used a mouse model where *Lgl1* knockout is targeted to NG2 cells^28^. We bred *NG2-Cre*^*ERT2*^ driver mice^9^ with red-fluorescence protein Cre-reporter (*Ai14-Tom*)^29^ and *Lgl1* floxed (*Lgl1*^*fl/fl*^)^21,23^ mice, as well as Lgl1 wildtype (*Lgl1*^*wt/wt*^) mice as controls. Compound transgenic mice were injected with tamoxifen once at P30 and recombined cells were identified by immunofluorescence staining for red fluorescent protein dTomato (aka Tom + cells) (Fig. 2a). NG2 and CC1 co-staining (Fig. 2b) revealed that amongst Tom+ cells, NG2 + CC1+ cells were increased (WT = 14.7% vs. cKO = 30.3%; *p* < 0.01), CC1+ cells were decreased (WT = 51.8% vs. cKO = 37.6%; *p* < 0.01) and NG2+ cells were not significantly changed in *Lgl1* cKO vs. *Lgl1* WT CC (Fig. 2c). These data show that Lgl1 is not required for CC1 expression in OPC progeny. Lgl1 is rather required for NG2 protein down-regulation and positively regulates OL differentiation.

To provide further evidence that Lgl1 positively regulates OL differentiation, we analyzed ex vivo isolated *Lgl1* cKO cells (Fig. 2d) for expression of OL maturation marker O4 and OPC marker PDGFRα at P60 (Figs. 2e, f, Supplementary Figs. 2a, b) and at P120 (Supplementary Figs. 2c, d). Indeed, we find that the total number of OPC increased to a greater extent than OL (OPC = 4.5 fold cKO vs. WT; *p* = 0.002; OL = 2.3 fold cKO vs. WT; *p* = 0.06) at P60 (Fig. 2e). Accordingly, the frequency of OPC amongst Tom +, recombined cells increased in *Lgl1* cKO, while the frequency of OL decreased amongst Tom+ cells (OPC/WT = 62% vs. OPC/cKO = 79%; OL/WT = 33% vs. OL/cKO = 19%; Fig. 2f). At P120, the frequency of OPC was still higher amongst *Lgl1* cKO Tom+ cells than amongst *Lgl1* WT Tom+ cells (OPC/cKO = 41.3% vs. OPC/WT = 23.4%; *p* = 0.02) and the frequency of OL was still lower (cKO = 39.4% vs. WT = 51.7%; *p* = 0.02; Supplementary Fig. 2d).

Taken together, these data show that upon *Lgl1* knockout, OL aberrantly retain NG2 protein expression and expand as OPC marker positive cells, while failing to further differentiate and mature.

In contrast to postnatal and adult OPC, embryonic OPC are not committed to the oligodendrocyte lineage and reportedly also generate astrocytes or neurons^9,30,31^. To test if *Lgl1* deletion affects the lineage commitment in young adult OPC, we quantified expression of neuroblast and astrocyte markers CD24 and GLAST, respectively, by flow cytometry (Supplementary Figs. 2a, b). As expected, only a small fraction of recombined cells expressed neuroblast and astrocyte markers (<2%) and the frequency of neuroblasts and astrocytes did not change significantly following Lgl1 deletion (Supplementary Fig. 2b). We concluded that Lgl1 loss does not alter the potential of young adult OPC to generate astrocytes and neuroblasts, suggesting a specific role of Lgl1 in OL differentiation.

Demyelinating injury increases the rate of OL proliferation and differentiation when compared with non-lesioned murine brain^32,33^. To test if Lgl1 is also required for OL differentiation in demyelinating lesions, we quantified the frequency of mature OL marker Plp in lysolecithin-induced lesions in *NG2-Cre*^*ERT2*^ *Ai14-TdTom Lgl1*^*fl/fl*^ and *Lgl1*^*wt/wt*^ mice injected with tamoxifen at P52 (Supplementary Figs. 2e, f). The frequency of Plp-positive cells amongst recombined cells was significantly decreased upon *Lgl1* deletion (WT = 73.3% vs. cKO = 33.0%; *p* < 0.01; Supplementary Fig. 2g), showing that Lgl1 is critical for OL differentiation in demyelinating lesions.

Lastly, we tested if the requirement for Lgl1 in differentiation can be recapitulated ex vivo. To this end, we isolated OPC from the corpus callosum of GFAP-Cre *Lgl1* cKO and *GFAP-Cre Lgl1* WT control mice and cultured them under OL differentiation conditions in vitro^34^ and stained cells for NG2 and O4. Quantifications revealed a significant increase of NG2+ cells and a decrease of O4+ cells amongst all Tom+ cells, upon *Lgl1* knockout (Figs. 2g, h).

Taken together, we conclude that Lgl1 is required for the downregulation of NG2 and OL differentiation.

### Lgl1 is required for asymmetric OPC divisions

**Fig. 3:**
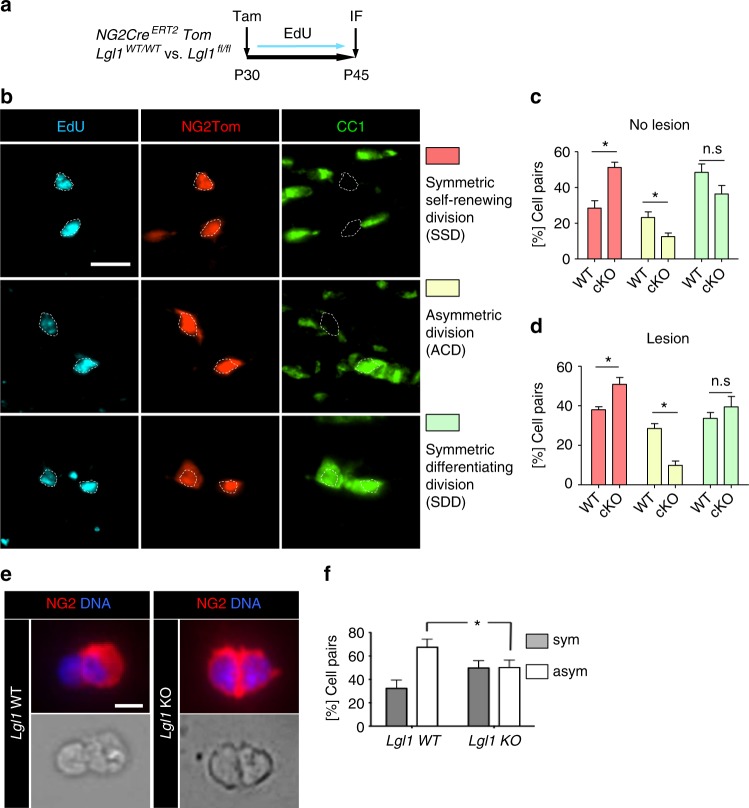
Lgl1 regulates asymmetric cell divisions of OPC. **a** Treatment schematic for in vivo pair assay in P30 *NG2-Cre*^*ERT2*^ *Tom Lgl1* WT vs. *Lgl1* cKO mice, which were given EdU for 14 days in drinking water. **b** Left panels: Representative cell pairs in the corpus callosum of treated mice and co-immunostained for red fluorescent protein (Tom) to label NG2+ cells and progeny, proliferation marker EdU and CC1. Symmetric, self-renewing divisions = two Tom+EdU+CC1− progeny, asymmetric cell division = one Tom+EdU+CC1− and one Tom+EdU+CC1+ progeny and symmetric, differentiating division = two Tom+EdU+CC1+ progeny. Scale bar: 10 µm. **c** Graph depicting the frequency of each cell division mode and showing a significant decrease of asymmetric divisions (yellow bars) and an increase of symmetric, self-renewing divisions (red bars) in *Lgl1* knockout (cKO) mice when compared with wildtype (WT) mice. Data are represented as mean ± s.e.m. from the analyses of 4 *Lgl1* WT vs. 4 *Lgl1* cKO treated mice (*n* = 4, *p < 0.05, Mann–Whitney test). **d** Graph depicting the percentage of three division modes during remyelination in *Lgl1* WT and *Lgl1* cKO corpus callosum, and showing a significant decrease of asymmetric divisions (yellow bars) and increase of symmetric, self-renewing divisions (red bars). Data are represented as mean ± s.e.m. from the analyses of 4 *Lgl1* WT vs. 4 *Lgl1* cKO treated mice (*n* = 4, *p < 0.05, Mann–Whitney test). **e** Representative NG2+ OPC pairs stained for NG2 (red) and DNA (blue) showing an asymmetric division in *Lgl1* WT (left panel) and symmetric, self-renewing division in *Lgl1* cKO (right panel) cells. Scale bar = 10 μM. **f** Graph depicting percentage of cell pairs and showing that Lgl1 loss increases symmetric, self-renewing divisions at the expense of asymmetric divisions in vitro (*n* = 6, *p < 0.05, Mann–Whitney test)

Postnatal and adult OPC divide asymmetrically to generate at a one-to-one ratio an NG2+ OPC and a CC1+ OL^2,3,10^. Given that *Lgl1* knockout reduces the number of CC1+ cells (Fig. 2c), we hypothesize that Lgl1 regulates ACD. To test this hypothesis, we visualized and quantified ACD in *Lgl1 NG2-Cre*^*ERT2*^ *Ai14-TdTom Lgl1*^*fl/fl*^ and *Lgl1*^*wt/wt*^ mice that were injected with tamoxifen once at postnatal day 30 (P30) for clonal recombination^35^. To monitor proliferation, mice were treated with EdU for 14 days (Fig. 3a), and cell pairs were identified by co-immunostaining for Tom, CC1, and EdU^2,3^ (Fig. 3b). Cell pairs in the corpus callosum were scored as (i) symmetric, self-renewing (both cells = CC1 negative), (ii) asymmetric (one CC1-positive and one CC1-negative cell) and as (iii) symmetric differentiating (both cells = CC1 positive; Fig. 3b). The average cell cycle length of OPC increases from ~3 days in P20 to 9 days in P60 animals^1^. Assuming a linear increase in cell cycle lengths between P30 and P45, recombined OPC should have divided two to three times during this period. Indeed, we were able to detect recombined cell pairs at P45. Amongst these, ACD rates were reduced in the *Lgl1* cKO (WT = 23.2% vs. cKO = 12.5%; *p* = 0.023). Vice versa, symmetric, self-renewing divisions were increased in the *Lgl1* cKO (WT = 28.4% vs. cKO = 51.2%; *p* = 0.028). Noteworthy, symmetric differentiated (CC1+) cell pair rates were not significantly changed upon *Lgl1* cKO (WT = 48.4% vs. cKO = 36%; *p* = 0.2) (Fig. 3c and Supplementary Table 1).

Similarly, cell pairs in lysolecithin-induced demyelinating lesions in the corpus callosum and spinal cord, showed increased rates of symmetric self-renewing divisions at the expense of ACD upon Lgl1 knockout (Fig. 3d and Supplementary Figs. 3a, b and Supplementary Table 1).

To test for a disruption of ACD ex vivo, we generated *Lgl1* KO and *Lgl1* WT control OPC and plated them for pair assays, as described (Fig. 3e)^2^. We found that the frequency of ACD was reduced in *Lgl1* KO OPC (WT = 72% vs. cKO = 49%) (Fig. 3f; *p* = 0.05).

These data show that Lgl1 does not prevent CC1 expression, but rather prevents downregulation of OPC marker NG2, thereby lowering rates of asymmetric distribution of NG2. Thus, Lgl1 is required for ACD and restricts symmetric, self-renewing divisions in the healthy and injured brain and in the injured spinal cord.

### *Lgl1* knockout increases OPC proliferation and promotes gliomagenesis

**Fig. 4:**
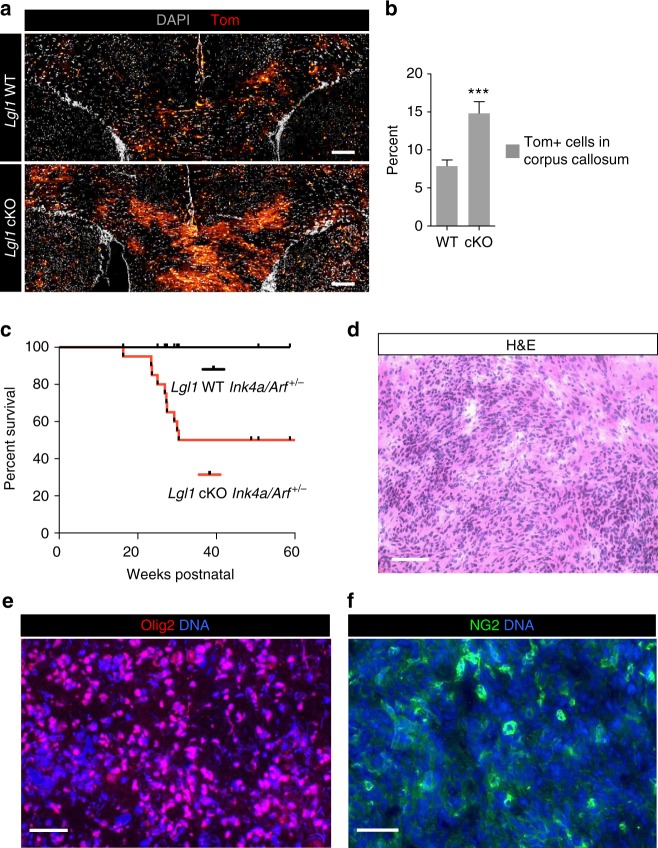
*Lgl1* knockout increases proliferation and promotes gliomagenesis. **a** Representative immunostaining of corpus callosum of *Lgl1* WT mouse (top panel) and Lgl1 cKO mouse (bottom panel) for Tom (red) and co-staining for DNA (gray) after broad deletion of Lgl1 in *NG2-Cre*^*ERT2*^ *Tom Lgl1 WT* (top panel) vs. *Lgl1* cKO P30 mice followed by analyses at P60 by FACS and IF. Scale bar: 200 µm. **b** Quantification of Tom+ cells by FACS from *Lgl1* WT and *Lgl1* cKO corpus callosum, showing a significant increase following Lgl1 knockout. Data are represented as mean ± s.e.m. from the analyses of 5 *Lgl1* WT vs. 5 *Lgl1* cKO mice (***p < 0.001, Mann–Whitney test). **c** Kaplan– Meier survival curve for *Lgl1* WT (*Lgl1*^*wt/wt*^*; NG2Cre; Ink4a/Arf*^*+/-*^) and NG2Cre-driven homozygous *Lgl1* cKO (*Lgl1*^*fl/fl*^*; NG2Cre; Ink4a/Arf*^*+/-*^) mice. Six animals were analyzed for each genotype. **d** Hematoxylin and Eosin (H&E) staining of endogenous tumors in *Lgl1* cKO mice, showing infiltrative growth. Scale bar: 100 μm. **e**, **f** Immunostaining of endogenous tumors in *Lgl1 NG2-Cre* mice for Olig2 (blue, **e**) and NG2 (green, **f**). Scale bar: 50 μm

Given that *Lgl1* knockout cells fail to restrict expression of pro-mitotic NG2, we hypothesize that their proliferation rates might be increased. We therefore assessed proliferation in *NG2-Cre*^*ERT2*^ *Ai14-TdTom Lgl1*^*fl/fl*^ and *Lgl1*^*wt/wt*^ mice injected with tamoxifen at P30 and exposed to EdU at P45 (Supplementary Figs. 4a–c) and at P60 (Figs. 4a, b and Supplementary Figs. 4d–f). Quantifications revealed that the overall percentage of Tom+ cells at P60 was significantly increased in the *Lgl1* cKO (14.8%) vs. the *Lgl1* WT corpus callosum (7.9%; *p* = 0.0005; Fig. 4a, b). Moreover, we chemically labeled EdU-positive (Edu+) cells. Edu+ cells were 2.3-fold more frequent amongst the Tom+ cells in *Lgl1* cKO vs. the *Lgl1* WT corpus callosum (*p* = 0.014; Supplementary Figs. 4b, c) at P45. A similar increase was observed in demyelinated corpus callosum of P55 mice (1.8-fold higher EdU+ cells amongst Tom+ *Lgl1* cKO vs. *Lgl1* WT; *p* = 0.007) (Supplementary Figs. 4d–f). These data indicate that Lgl1 restricts proliferation of OPC in the healthy and demyelinated CC.

Increased proliferation, defective differentiation, and decreased rates of ACD are associated with gliomagenesis^2^. We therefore tested whether *Lgl1* knockout promotes tumor formation. Immunostainings of normal adult mouse brain revealed that OL express the tumor suppressor p19^Arf^ (Supplementary Fig. 5a)^36^. We therefore bred Lgl1 floxed mice^21^ with a *NG2-Cre* driver line^37^, and mice heterozygous for the *Ink4a/Arf* deletion (*Ink4a/Arf*+*/*−)^38^. Mice were monitored for neurological signs of tumor formation and survival. We observed that 100% of *Lgl1* WT *Ink4a/Arf*+*/*− mice were still alive at 60 weeks postnatal age, whereas *Lgl1* cKO *Ink4a/Arf*+*/*− mice had a significantly shorter median survival of 45.79 weeks (Fig. 4c; *p* = 0.0108). Histopathologic analyses of *Lgl1* cKO *Ink4a/Arf*+*/*− brains showed infiltrative tumors consistent with astrocytoma morphology (Fig. 4d). Expression analysis by qrt-PCR in tumor cells isolates showed a strong reduction of *p19*^*Arf*^ mRNA versus *Lgl1* cKO *Ink4a/Arf* WT and *Ink4a/Arf*+*/−* cells, suggesting that the WT allele of *p19*^*Arf*^ is not expressed in the tumor (Supplementary Fig. 5b). Fluorescent immunostainings of brain sections from tumor-bearing mice showed that tumors express the oligodendrocyte lineage and glioma markers Olig2 and NG2. Results are indicative of a mixed astrocytic and oligodendroglial high-grade glioma (Figs. 4d–f)^39^.

To determine whether our animal model findings were clinically relevant, we mined expression data from 99 patients from The Cancer Genome Atlas (TCGA) for a potential association of Lgl1 mRNA expression levels and glioblastoma patient survival. We found that low levels of Lgl1 mRNA expression correlated with poor patient survival (Supplementary Fig. 5c)^40^. In further support of a tumor suppressive function, Lgl1 protein expression was absent in six out of seven human glioblastoma xenografts as shown by immunoblotting (Supplementary Fig. 5d). Collectively, these data showed that Lgl1 attenuates OPC proliferation and synergizes with tumor suppressor Ink4a/Arf to suppress gliomagenesis.

### *Lgl1* cKO OPC show a defect in NG2 routing to the lysosome

**Fig. 5:**
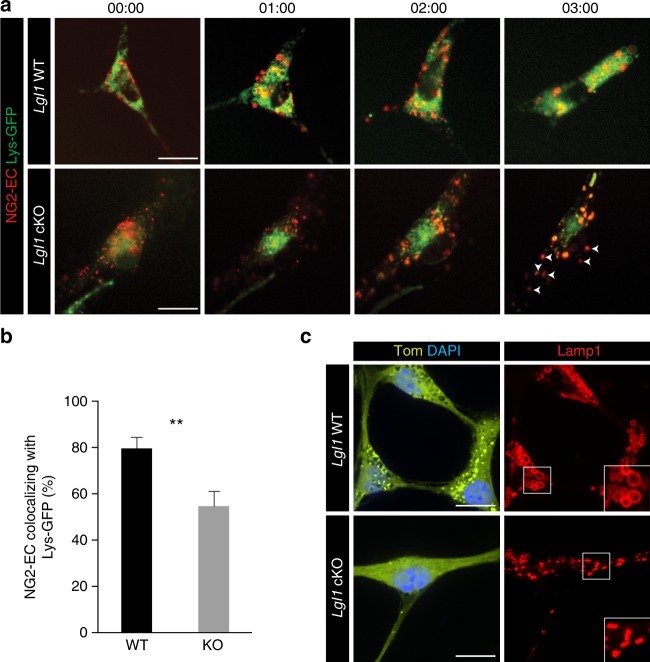
*Lgl1* cKO OPC show defect in NG2 routing to the lysosome. **a** Time-lapse fluorescent pictures of *Lgl1* WT and *Lgl1* cKO OPC labeled for NG2-EC (red) and lysosome (Lys-GFP, green) under differentiation conditions. Pictures are acquired every hour for 3  hours. White arrows indicate NG2-EC that did not reach the lysosome. Time indicated as hours:minutes. Scale bar: 25 μm. **b** Quantitative graph representing the ratio of labeled NG2 puncta in the lysosomes in WT (black) and *Lgl1* cKO cells (gray). Data are represented as mean ± s.e.m., *n* = 10 cells each genotype, three independent experiments (**p < 0.01, Student’s *t*-test). **c** Immunostaining of *NG2dTom* cells (yellow) with lysosomal protein Lamp1 (red) showing the difference in lysosome shape between *Lgl1* WT OPC and cKO OPC. Scale bar: 25 μm

A recent study showed that levels of surface NG2 are regulated by endocytosis^15^. We hypothesize that *Lgl1* cKO OPC fail to route NG2 to the lysosome and thereby sustain aberrantly high NG2 levels. In order to test this, we transfected *Lgl1* cKO and WT cells with lysotracker GFP (Lys-GFP) and labeled cell surface NG2 with an antibody specific for its extracellular domain (NG2-EC) and a fluorescent secondary antibody. Cells were consecutively monitored by fluorescent time-lapse microscopy for up to three hours under differentiation conditions. In both *Lgl1* cKO and WT OPC, NG2-EC started to reach the lysosome one hour post imaging (Fig. 5a). Co-localization of NG2-EC with Lys-GFP peaked in *Lgl1* WT cells three hours after starting the imaging. Interestingly, we observed a significantly higher colocalization of NG2 with lysosomes in WT OPC compared to *Lgl1* cKO OPC (WT = 79.6%; cKO = 54.7%, *p* = 0.0047) (Fig. 5a) suggesting that in differentiating OPC, Lgl1 acts as a positive regulator of NG2 trafficking to the lysosome. These results prompted us to stain for the lysosome associated membrane protein 1 (Lamp1) to investigate whether the lysosome is formed properly in *Lgl1* knockout cells. We find that lysosomes are present in *Lgl1* cKO OPC, based on Lamp1 positivity and subcellular localization. Noteworthy, lysosome shape appears to be altered due to *Lgl1* knockout (Fig. 5c). Collectively, these results show that Lgl1 positively regulates NG2 trafficking to the lysosome.

### Non-degraded NG2 in *Lgl1* cKO OPC is recycled to the membrane

**Fig. 6:**
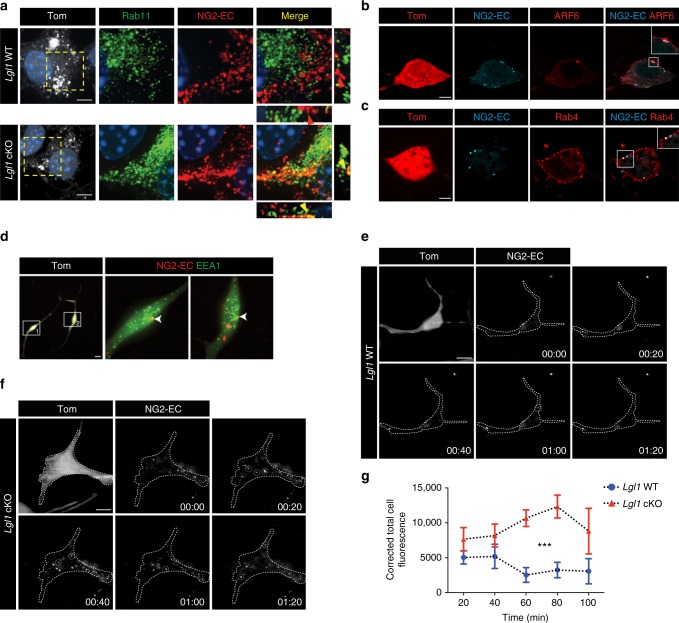
Non-degraded NG2 in *Lgl1* cKO OPC is recycled to the membrane. **a** Representative co-immunostaining for NG2-EC (red) and Rab11 (green) 45 min after incubation in differentiation medium. Note the higher colocalization of NG2-EC with Rab11 in *Lgl1* cKO OPC (yellow arrowheads) compared to *Lgl1* WT cells (red arrowhead). Scale bar: 5 μm. **b**–**d** Fluorescent immunostaining pictures illustrating colocalization of remaining NG2 (blue) with (**b**) ARF6 (red), (**c**) Rab4 (red), and (**d**) EEA1 (green) in Lgl1 cKO cells 24 hours after incubation in differentiation conditions. Scale bars: 10 μm. **e**, **f** Frames illustrating TIRF time lapse of *Lgl1* cKO and WT cells 4 hours after incubation in differentiation conditions. Pictures were taken each 20 min. Examples of recycling labeled NG2 puncta (white) are highlighted in dashed circle. NG2 recycling persists in *Lgl1* cKO cells up to time point 01:20 whereas it is insignificant in WT cells. Time indicated as hours:minutes. Scale bar: 10 μm. **g** Graph representing quantification of the corrected total cell fluorescence reflecting NG2 recycling in *Lgl1* cKO cells (blue circles) and in *Lgl1* WT cells (red lozenges). Data are depicted as mean ± s.e.m., *n* = 8 cells for each genotype, three independent experiments (***p < 0.01, two-way ANOVA)

Given that NG2 is a transmembrane proteoglycan^41^, we suggest that it might be recycled to the membrane by the recycling endosome, a tubular structure continuous with the early endosome. To test this, we incubated *Lgl1* cKO cells in differentiating conditions and live labeled NG2-EC. Cells were fixed at different time points after surface NG2 labeling and stained for recycling protein markers. At 45 min, we observed a higher colocalization of NG2-EC with the recycling regulator Rab11 in *Lgl1* cKO compared to WT cells (Fig. 6a). At 24 h, NG2-EC colocalizes with ARF6, a GTPase critical for endocytic recycling, supporting the notion that NG2 is recycled to the membrane in *Lgl1* cKO OPC (Fig. 6b). Additionally, NG2-EC overlaps with Rab4, a GTPase marker for fast endocytic recycling, hinting that NG2 recycling may not be restricted to the slow recycling pathway (Fig. 6c). We also observed co-localization of NG2-EC with the early endosome marker EEA1 (Fig. 6d), suggesting that NG2 is constantly uptaken and shuffled back to the membrane. Noteworthy, we observed a coexpression of Lgl1 and the early endosome-specific protein Rab10 both at the membrane and around the nucleus, further associating Lgl1 role with the endocytic machinery^42^ (Supplementary Fig. 6).

In order to better investigate a potential NG2 recycling in *Lgl1* cKO cells, we used total internal reflection fluorescence (TIRF) to monitor live-labeled NG2-EC docking to the membrane. Cells were plated in differentiation medium for four hours and then monitored. By this time, the majority of NG2 is degraded in *Lgl1* WT cells and no significant NG2 recycling was observed (Fig. 6e,). To the contrary, NG2-EC was more abundant and continuously re-introduced to the membrane in *Lgl1* cKO OPC (Figs. 6f, g; Supplementary Movie 1 see Methods).

Collectively, these data proof that in *Lgl1* cKO cells, NG2 is mostly recycled to the membrane rather than degraded.

### *Lgl1* cKO OPC show premature and accelerated endocytosis recycling of NG2

**Fig. 7:**
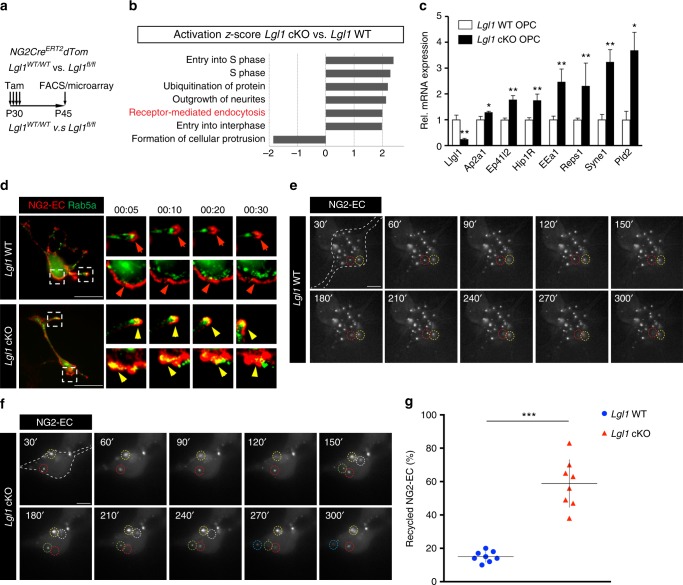
Lgl1 negatively regulates internalization and recycling of NG2 in OPC. **a** Schematic illustration of the experimental approach assessing global changes in gene expression by microarray in *Lgl1* WT vs. *Lgl1* cKO OPC. FACS sorted red fluorescent PDGFRα+O4− cells. **b** Bar graph of ingenuity pathway analyses (IPA) of *Lgl1* WT vs. cKO OPC. Receptor-mediated endocytosis pathway is amongst the upregulated pathways in *Lgl1* cKO cells. **c** Validation by q-RT-PCR for mRNA encoding receptor-mediated endocytosis regulators that were differentially expressed in *Lgl1* cKO cells as determined by microarray analyses. OPC were derived from the corpus callosum of *GFAP-Cre*, *Tom*, *Lgl1*^*wt/wt*^ and, *GFAP-Cre*, *Tom*, *Lgl1*^*fl/fl*^ mice. Data represented as mean ±s.e.m. *n* = 6 *Lgl1* WT and 6 *Lgl1* cKO animals (*p < 0.05, **p < 0.01, Mann–Whitney test). **d** Steady-state frames of time lapse (1 frame/ 10 min) of NG2-EC (red) and early-endosome marker Rab5a (green) in *Lgl1* cKO and WT OPC. Note that NG2 stays mostly at the membrane in *Lgl1* WT cells (red arrows), whereas it co-localizes with the early endosome in the cytoplasm of *Lgl1* cKO OPC (yellow arrows). Scale bar: 20 µm. **e**, **f** Frames illustrating time lapse experiment under TIRF microscopy. Pictures are acquired each 30 seconds as soon as cells were exposed to differentiation conditions. Each white puncta in focus represents labeled NG2-EC docked at the membrane. Individual recycled puncta are highlighted with dashed circles of different colors. Note that in *Lgl1* cKO cells, the majority of the labeled NG2 is internalized and recycled to the membrane whereas in WT cells, the majority of NG2 puncta retains a stable localization at the membrane. Scale bars: 10 μm. **g** Graph representing the quantification of NG2 recycling at the membrane throughout the first 5 min of differentiation conditions. Data represented as mean ± s.e.m. *n* = 3 cells per genotype, three independent experiments (***p < 0.005, Student's *t*-test)

To better characterize the deregulation of endocytosis in *Lgl1* knockout cells, we acutely isolated Tom+ cells from Lgl1 *NG2-Cre*^*ERT2*^ *Ai14-TdTom Lgl1*^*fl/fl*^ and *Lgl1*^*wt/wt*^ control mice injected with tamoxifen at P30. Fifteen days after starting *Lgl1* knockout, gene expression was analyzed using high-sensitivity, whole-transcript arrays (Fig. 7a; see also Methods). By analyzing the 318 genes differentially expressed between *Lgl1* WT and *Lgl1* cKO OPC (*p* < 0.05), ingenuity pathway analysis identified several pathways including the receptor-mediated endocytosis pathway to be upregulated in *Lgl1* cKO cells (Fig. 7b; activation *z*-score = 2, *p* = 0.018). Upregulation of early endocytosis genes *Ap2a1*, *Ep41I2*, *Hip1R*, *EEa1*, *Reps1*, *Syne1*, and *Pdl1* was further validated by qrt-PCR in *Lgl1* cKO OPC vs. *Lgl1* WT OPC (Fig. 7c). These data suggest that Lgl1 knockout deregulates endocytic uptake in addition to endocytic recycling.

In order to investigate if early endocytosis is affected in *Lgl1* cKO cells, we labeled surface NG2 with the NG2-EC antibody, and marked the early endosome by expression of Rab5a-GFP. Time-lapse imaging of cells for 30 min in differentiation conditions showed a prominent colocalization of NG2 with Rab5a only in mutant cells, suggesting that Lgl1 negatively regulates NG2 uptake (Fig. 7d). We corroborated these results by quantifying the endocytosis of transferrin using FACS, and found a ten-fold increase in transferrin incorporation in *Lgl1* cKO OPC as compared to *Lgl1* WT OPC (Supplementary Fig. 7).

Next, we tested whether NG2 is only recycled after failing to be degraded or also when prematurely internalized in *Lgl1* cKO OPC. We used TIRF to monitor labeled NG2 docking to the membrane over the first 5 min of differentiation conditions. We observed that a significantly higher ratio of labeled NG2 was endocytosed and recycled to the membrane in *Lgl1* cKO cells compared to WT cells (WT = 15%; cKO = 58%, *p* < 0.0001) (Figs. 7e–g). Taken together, these data show that Lgl1 negatively regulates NG2 internalization and recycling and establish Lgl1 as a regulator of NG2 trafficking by the endocytic pathway.

### Differentiation defect in *Lgl1* cKO OPC is linked to aberrant NG2 recycling

**Fig. 8:**
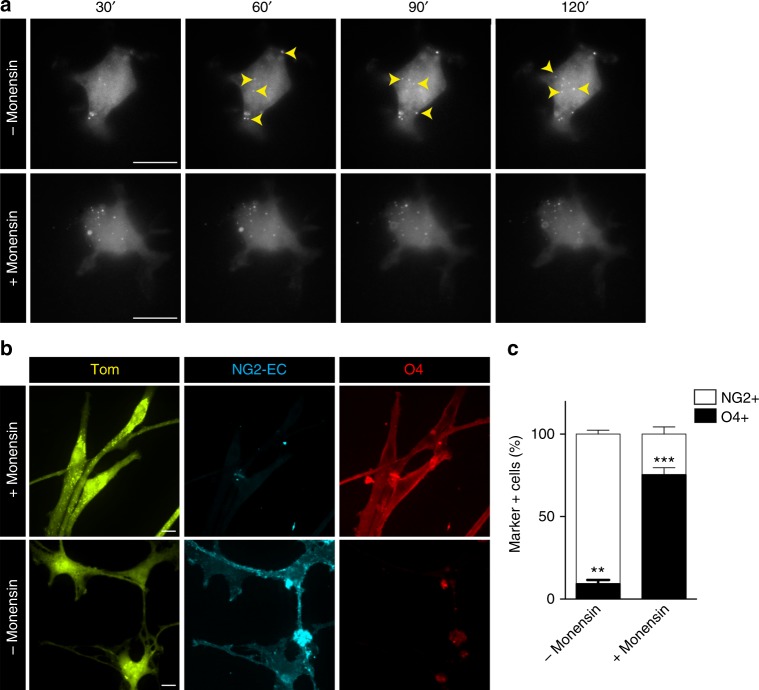
Aberrant NG2 recycling is linked to differentiation defects. **a** Steady-state images of two single *Lgl1* cKO cells monitored for NG2-EC (white dots) by TIRF, treated (bottom panel) or not treated (upper panel) with monensin. Pictures are acquired each 30 seconds. Note that NG2 is recycled to the membrane in non-treated cells (yellow arrow), whereas no recycling is observed in treated cells. Scale bar: 10 μm. **b** Fluorescent pictures illustrating *Lgl1* cKO cells (Tom) monensin -treated or untreated and labeled for NG2 (blue) and O4 (red) after 5 days in differentiation conditions. Scale bar: 10 μm. **c** Graph representing quantification of O4+ and NG2+ cells in monensin-treated or untreated *Lgl1* cKO cells. Untreated cells are mostly NG2+ and O4−, whereas treated cells show a reverse phenotype with a higher number of cells expressing O4. Data are depicted as mean ± s.e.m., *n* = 20 cells per genotype, three independent experiments (**p < 0.01, Student's *t*-test)

Our studies above suggest that *Lgl1* knockout enhances NG2 internalization and recycling to the membrane, thereby preventing NG2 degradation and blocking OL differentiation. In order to dissect whether abnormal NG2 recycling has an effect on OL differentiation, *Lgl1* cKO cells were incubated for 4 hours with monensin (10 μM), a drug widely used to interrupt protein recycling^43^. We tested if the drug has a specific effect on NG2 recycling in *Lgl1* cKO cells by monitoring Tom+ cells under TIRF for up to one hour after monensin treatment. We observed that monensin treatment blocked NG2 recycling, validating the reported drug effect on recycling (Fig. 8a)^43^. Lastly, we assessed differentiation in treated and non-treated *Lgl1* cKO cells kept in differentiation conditions for 5 days by O4 and NG2 co-immunostaining. A quantification of marker-positive cells showed that monensin treatment significantly increased the ratio of O4+ cells at the expense of NG2+ cells (treated/O4+ = 75% and NG2+ = 24%; non-treated/O4+ = 9% and NG2+ = 90%; *p* < 0.0001) (Figs. 8b, c).

These results link abnormal recycling of surface NG2 and defective OL differentiation.

## Discussion

Adult OPC differentiate in multiple steps, beginning by committing to OL fate and ending by maturing and producing myelin. There is a wealth of studies on the later steps of OL differentiation and maturation^34,44^. Little is known, however, about the earliest decisions between OPC and OL fate. Thus far, the down-regulation of proteoglycan NG2 is the earliest immunophenotypic change that distinguishes the two daughter cells within a newly formed OPC pair upon ACD. NG2 marks the OPC daughter, while the NG2-negative daughter acquires CC1 expression, which marks OL differentiation^2,3,9^. NG2 knockout OPC show delayed proliferation, which indicates that NG2 controls the timing of OPC proliferation^7^ (reviewed by Sakry et al.^45^). The NG2 knockout does not fully block OPC proliferation and differentiation and its effect is only temporary^7^. Thus, downregulation of NG2 is an important but not the only step for OL commitment. Indeed, PDGFRα is also asymmetrically distributed amongst newly formed OPC pairs^3^, further suggesting that cell fate determinants in addition to NG2 link ACD to differentiation^46^.

Due to the reported functional, morphologic, and neuroanatomical heterogeneity of OPC^47^, and its potential influence on cell division mode, cell cycle lengths, and differentiation^1,9,48,49^, we focused our studies on OPC in the corpus callosum. We analyzed OPC in mice between the ages P30 and P60 with the exception of tumor-bearing mice, which were older. The rationale for analyzing OPC at these stages was that the analyses of mice older than P60 would be technically very difficult due to a long OPC cell cycle time^1^. We expect to detect the maximum number of ACD between P30 and P60, because the rates of asymmetric OPC divisions are already high at postnatal stages (P20; ACD = ~35%) and peak at P60 (ACD = ~45%) before decreasing again with age (P120; ACD = ~35%)^3^. Moreover, the rate of differentiation remains stable at 40% between P30 and P60^9^, which makes is a suitable range to identify potential effects on differentiation of a single gene knockout.

Since ACD generate differentiated cells from stem and progenitor cells across species, it was attractive to speculate that conserved regulators of ACD, such as Lgl1, regulate early steps in OL differentiation. Previous studies showed that Lgl1 localizes to the lateral membranes and cytoplasm of embryonic neural progenitor cells^21,22^. Expression of Lgl1 protein in rat neocortex homogenates persists into adulthood^42^. Functional analyses in the adult brain, however, were limited to its role in SVZ neural stem cells, neurons, and astrocytes^21–23,42^.

Here, we show that *Lgl1* cKO cells fail to lose NG2 expression and exhibit differentiation defects. Interestingly, we find that *Lgl1* knockout in OPC increases the rates of cells in a transitional state and expressing both OPC marker NG2 and OL marker CC1, at the expense of mature OL. These data indicated that Lgl1 loss does not prevent the expression of CC1 but rather prevents downregulation of NG2, and thereby permanently attenuates differentiation.

Whereas normal OPC populate the brain sparsely and show little overlap in the areas that two adjacent OPCs occupy^50^, they are denser in demyelinated lesions, such as those typically found in chronic inflammation models in mice and in multiple sclerosis patients^32,33^. We found that Lgl1 is a critical regulator of OL differentiation during remyelination following chemical-induced demyelination. OPC are considered a critical source for remyelination and failed OPC differentiation are thought to be one important underlying cause for failed remyelination in chronically demyelinated lesions^51^. It is interesting to speculate that Lgl1 functions at an early step in the complex regulatory process of remyelination and thus may be a viable target for approaches aimed at promoting proper differentiation of OPCs in demyelinated lesions.

Our analyses of cell division modes support the hypothesis that Lgl1 restricts NG2 during ACD as part of the mechanism to turn on OL differentiation in the NG2-negative progeny. Moreover, Lgl1 does not disrupt symmetric, differentiating divisions, which suggests that it specifically regulates differentiation in the ACD progeny. To date, only a few conserved ACD regulators have a known function in OL differentiation in addition to Lgl1. These include the polarity regulator partitioning-defective 3 (Par3) protein, which promotes myelination in the peripheral nervous system^52^, and the transcriptional regulator Prox1, which is required for OL lineage in the mouse. Par3, Prox1, and Lgl1 are in the same protein network that controls ACD in *Drosophila* neuroblasts^46^. A role for Par3 and Prox1 in ACD of OPC has yet to be determined.

Multiple studies have shown that OPC^13,14^ and OL^36^ are a cellular origin of glioma in genetically engineered mouse models. The neoplastic transformation of OPC and OL and formation of gliomas are associated with increased proliferation, decreased differentiation, changes in the tumor microenvironment^53^, and a decrease in ACD^2^. *Lgl1* knockout during embryonic neurogenesis disrupts the asymmetric division of neuroepithelial cells and generates rosette-like structures reminiscent of PNETs but perinatal death prevented the analyses of tumor formation^21^. *Lgl1* knockout in cell types known to only undergo symmetric but not asymmetric divisions, such as astrocytes, did not generate premalignant lesions^23^. Taken together with our data, these findings suggest that defective ACD might be an underlying cause for proliferation and differentiation defects in glioma cells. Our data support the notion previously observed in epithelial cells^54^ that loss of polarity—albeit by itself not sufficient to induce carcinogenesis—contributes to tumor formation. We show that Lgl1 loss synergizes with Ink4a/Arf loss, which is a frequent and early event in glioma^24^, to induce gliomagenesis (Fig. 4). There is strong precedence for *Ink4a/Arf* heterozygous mice to be as susceptible as *Ink4a/Arf* nullizygous mice to gliomagenesis^55^. A likely explanation for this is, that the remaining Ink4a/Arf allele in tumors arising from *Ink4a/Arf*+*/−* mice is inactivated at high frequency, which is supported by our findings that p19^Arf^ mRNA expression is greatly reduced in *Lgl1* KO tumors. Although 19^Arf^ and p16^Ink^ transcripts are reportedly low in OPC and OL, we find overlapping expression of the p1*9*^*Arf*^ protein and OL marker CC1 (Supplementary Fig. 5). This is interesting, because p19^Arf^ but not p16^Ink^ is an important tumor suppressor in a glioma mouse model study^36^. Collectively, the data suggest that tumor formation in *NG2-Cre Lgl1* KO *Ink4a/Arf*+*/−* mice required inactivation of p19^Arf^ expression, hence the long latency (Supplementary Fig. 5). It is also plausible that Lgl1 suppresses tumor formation by restricting NG2 function as a co-receptor for receptor tyrosine kinases, since deregulated PDGFRα and EGFR signaling activity contributes to gliomagenesis^40,56^ (for review see ref. ^57^).

Endocytosis has a range of functions including regulating the cell surface expression and subcellular localization of proteins, remodeling the membrane and controlling signaling pathways^16^. Studies in *Drosophila* epithelia cells support a link of polarized protein localization and endocytosis by showing that loss-of-function mutants of the critical endosome adaptor AP2 leads to defects in polarity and differentiation similar to those observed in Lgl loss-of-function experiments^58^. Our study proposes an unprecedented link between defects in NG2 endocytic trafficking, ACD, and differentiation. Indeed, in *Lgl1* cKO mutant cells, we find that NG2 is not properly routed to the lysosome and instead is recycled to the membrane. These observations show that endocytic pathway defects in *Lgl1* cKO OPC disturb the regular intracellular itinerary of NG2 during differentiation and lead to its recycling rather than degradation. NG2 levels are permanently suppressed in OL most likely as a result of downregulation of the NG2 promoter in the OL daughter after ACD^3^.

Taken together, these data suggest that the unequal distribution of NG2 in the newly formed progeny of ACD is achieved by NG2 degradation in the lysosome and is maintained by silencing the NG2 promoter in the OL daughter.

We can only speculate as to whether Lgl1 regulates NG2 trafficking directly or, indirectly, by regulating the endocytic machinery. Although not previously implicated as a negative regulator of endocytosis in mammalian cells, Lgl1 activates endocytosis protein and GTPase Rab10 by releasing the GDP dissociation inhibitor (GDI) in rat hippocampus to induce polarized axon growth^42^. In cells undergoing ACD, such as *Drosophila* neuroblasts, Lgl1 establishes cell polarity by mechanisms that regulate myosin II activity, directed vesicle transport and the subcellular localization of proteins (for review see ref. ^46^). Nonmuscle myosin II (NMII) is a critical regulator of clathrin-mediated endocytosis^59^ and is a negative regulator of oligodendrocyte morphological differentiation^60^. Lgl1 associates with NMII and inhibits filament formation^61^ and reduces the association of NMII with actin. Whether Lgl1 regulates endocytosis by affecting motor protein dynamics or through other mechanisms, such as regulating the subcellular localization of the GTPases^62,63^, remains to be determined. In further support of an indirect effect of Lgl1 on endocytic trafficking, *Drosophila* Lgl1 regulates vesicle acidification in the lysosome^64,65^. Noteworthy, our data indicate that lysosomes in *Lgl1* cKO OPC have a tubular shape rather than the vesicular shape seen in WT OPC, hinting that lysosome maturation might be affected by *Lgl1* knockout (Fig. 5).

Future studies should investigate further the potential link of defects in endocytosis and gliomagenesis.

## Methods

### Mice

All mouse experiments were approved by and performed according to the guidelines of the Institutional Animal Care and Use Committee of the University of California, San Francisco. *Lgl1*^*lox/lox*^ mice were courtesy of Dr. Vasioukhin^21^ and were bred with *NG2-Cre* (Jax strain 008533)^37^ and *GFAP-Cre* (Jax strain 004600)^66^, *NG2-CreER*^*TM*^ (Jax strain 008583)^9^ and *Ai14 Cre* reporter mice (Jax strain 007908)^29^ mice or *Ink4a/Arf* hemizygous knockout mice^38^. *Ascl1GFP*^67^ and *NG2DsRed*^37^ mice from Jackson Laboratories were bred together to obtain *Ascl1GFP-NG2DsRed* double transgenic mice. FVB/N mice were obtained from Simonsen Laboratories, Gilroy. For survival studies, *Lgl1* cre-conditional deleted mice were kept under observation until reaching the major endpoint, which was animal neurological symptom-free survival.

### Induction of *Lgl1* knockout and demyelination

Tamoxifen (Sigma-Aldrich) was dissolved in corn oil (Sigma-Aldrich) at 20 mg/mL and injected intraperitoneally (i.p.) at 1 mg to induce Cre activity Lα-lysophosphatidylcholine (lysolecithin = LPC, Sigma-Aldrich) was dissolved at 1% in physiological serum and 0.5 μl were injected to induce demyelination. For clonal labeling of NG2 cells P30-P52 *NG2-Cre*^*ERT2*^, *TdTom, Lgl1*^*fl/fl*^ and *NG2-Cre*^*ERT2*^, *TdTom*, *Lgl1*^*wt/wt*^ mice were injected with 1 mg tamoxifen twice within 24 h^35^. To assess proliferation rates and cell division modes of OPC, mice were subsequently treated with 0.1 mg/mL EdU in drinking water for 14 days and resected brains were processed for immunofluorescence. For differentiation *NG2-Cre*^*ERT2*^, *TdTom, Lgl1*^*fl/fl*^ and *NG2-Cre*^*ERT2*^, *TdTom*, *Lgl1*^*wt/wt*^ mice were injected with tamoxifen either once or twice daily for 4 consecutive days. Mice were analyzed 30 days post injection.

For broader deletion of Lgl1, P30-P52 *NG2-Cre*^*ERT2*^, *TdTom*, *Lgl1*^*fl/fl*^ and *NG2-Cre*^*ERT2*^, *TdTom*, *Lgl1*^*wt/wt*^ mice were injected with tamoxifen twice daily for 4 consecutive days. FACS isolated OPC were subjected at P45 to microarray analyses. To assess proliferation and cell division modes in demyelinated lesions, demyelination was induced by a single LPC injection into the CC or the spinal cord of mice previously injected with tamoxifen. For differentiation, demyelination was induced by LPC injection after the last tamoxifen injection and mice were sacrificed 10 days after to evaluate the effect on OPC differentiation. For spinal cord white matter demyelination, mice were anesthetized with isofluorane and demyelination was induced by LPC into the ventral or dorsal white matter funiculus at the level of T12^68,69^. For CC demyelination, LPC was injected in the right or the left hemisphere (AP+1 mm, *L* ± 1 mm, DV-2.1 mm)^70^. For EdU labeling, mice were injected i.p. with 10 mg/mL EdU twice a day, or EdU was mixed with drinking water at a low concentration (0.1 mg/mL) for 5–14 days. For long-term tracking of Lgl1 deletion effects, tamoxifen was injected at P30 for 5 consecutive days, and tissue analyzed by FACS at P120.

### Cell culture

Cells were isolated from the CC, which includes the subcallosal zone of P30–P60 mice, and cultured as non-adherent spheres in proliferative medium with NT3, CNTF, PDGFA, and FGF2 (Peprotech)^34^. Differentiation was induced by plating cells on poly-l-Lysine coated glass-bottom 96-well plates (CORNING) at 5000 cells/mL in medium containing CNTF, FGF2, and T3^2^. Cells were incubated with inhibitor of endocytosis Pitstop1 at 25 μM. To induce Lgl1 deletion in vitro, cells were incubated with adenovirus-cre-GFP (Vector BioLabs) and adenovirus-GFP^2^. Viability assay was performed using the Alamar Blue reagents^71^.

### Pair assay

Cells were subjected to a pair assay by plating dissociated single cells at clonal density for 12–36 h. Cell pairs formed were fixed and subjected to immunostaining with primary antibody and fluorescence-coupled secondary antibody^2^. Nuclei were visualized using DAPI (Sigma). Unless indicated otherwise, for pair assay a minimum of 50 and up to 200 cell pairs per cell type and condition, respectively, were analyzed, using a fluorescent microscope.

### Immunofluorescence and in vivo cell pair assays

*NG2-Cre*^*ERT2*^,*TdTom*, *Lgl1*^*fl/fl*^ and *NG2-Cre*^*ERT2*^,*TdTom*, *Lgl1*^*wt/wt*^ mice injected with tamoxifen were PFA perfused, brains were resected and frozen, sectioned and post-fixed for 10 min with 4% PFA, blocked with PBS 5% normal goat serum (NGS) or normal donkey serum (NDS) and 0.1% Triton X-100 for 1 h. For cell pair assays, sections were stained for anti-red fluorescence antibody (to detect the NG2+ cells and progeny with Lgl1 deletion) and anti-CC1 antibody overnight, washed and stained with fluorescence-couple secondary antibodies. Cell pairs were identified as two red fluorescence-positive (NG2Tom) and EdU-positive cells spaced less than 20 µm^2,3^. The entire CC was counted as three or more distinct brain sections per animal in 4–5 individual animals per group. Cell pairs with one CC1-positive cell were scored as an ACD.

The following antibodies were used at the given concentration:

α-Rb NG2 (Millipore 1:200), αMs-IgM O4 (R&D, 1:400), α-Ms CC1 (Calbiochem, 1:100), α-Gt Dcx (Santa Cruz Biotechnology, 1:300), α-Gt Hugl-1 (Lgl1, Santa Cruz biotechnology, 1:200), α-Rb Hugl-1 (Lgl1, Santa Cruz Biotechnology, 1:200), α-Ms Nestin (Chemicon international, 1:400), α-Rb PlP (abcam105784, 1:1000), α-Rat MBP (abcam7349, 1:400), α-Rb-beta galactosidase (ab616, 1:200), gt-α-Olig2 (EMD Millipore; 1:200), α-Rb-Rab11 (Life Technologies Corporation, 1:200); α-Rb-p19Arf (Santa Cruz; 1:100); α-Gt-Lamp1 (R and D systems AF4320, 1:100); α-ARF 6 (Santa Cruz sc-7971; 1:200), α-Ms EEA1(Santa Cruz sc-365652, 1:150); α-Rb-Rab4 (Millipore 07-655; 1:200); α-Ms-Rab10 (Life Technologies Corporation, 1:100).

For O4 staining, cells were stained live in vitro: O4 was incubated 1 h (1:400) at 37 °C in the corresponding culture medium (OPC proliferation or OL differentiation) before PFA fixation and further antibody staining.

EdU was visualized using the Click-it reaction kit (Life Technologies Corporation) following the manufacturer’s instructions^71^.

Early endocytosis compartment was visualized using the BacMam technology following manufacturer’s instructions (Life Technologies Corporation). Immunofluorescent images were acquired on a Nikon Ti-E microscope with Nikon perfect focus system equipped with a Hamamatsu Flash4.0 camera equipped with a 40×/0.75 Plan Fluor objective and/or Nikon Ti Yokogawa CSU-22 spinning disk confocal equipped with a Plan Apo VC 60×/1.4 oil objective. Images were analyzed with NIS-Elements 4.2 (Build 982) and ImageJ.

### FACS

*NG2-Cre*^*ERT2*^,*TdTom*, *Lgl1*^*wt/wt*^ and, *NG2-Cre*^*ERT2*^,*TdTom*, *Lgl1*^*fl/fl*^ mice were analyzed by FACS 30 days or 120 days after tamoxifen injection at P30. Lateral ventricle walls and/or CC cells were isolated from Ascl1-GFP and NG2-DsRed double-transgenic mice at P60. For DNA content analysis, dissociated cells were incubated with the vital DNA marker Hoechst 33342 (Sigma)^72^. The antibodies to identify distinct cell populations were anti-O4–biotin (MACS Miltenyi Biotec; 1:10) conjugated to streptavidin–FITC (MACS Miltenyi Biotec; 1:10); CD140a phycoerythrin–cyanine7 [PC7]-conjugated (mouse clone APA5; 1:100 eBiosciences); CD24 phycoerythrin–cyanine7 [PC7]-conjugated (Rat IgG2b; 1:100 Life Technologies); CD140b [APC]-conjugated (life technologies clone APB5, 1:50), and Glast-APC (MACS miltenyi, 1:30). Cells were gated following the fluorescence minus one (FMO) control. For microarray analyses, CC tissue was isolated from *NG2-Cre*^*ERT2*^, *TdTom*, *Lgl1*^*fl/fl*^ (*n* = 4) and *NG2-Cre*^*ERT2*^, *TdTom*, *Lgl1*^*wt/wt*^ (*n* = 5) triple-transgenic, P30 mice injected twice daily with tamoxifen for 4 consecutive days and OPC were obtained by sorting for Tom-positive, PDGFRα-positive O4-negative cells.

To perform absolute cell counts, single cell suspensions were transferred to tubes containing a calibrated number of fluorescent beads (TruCount tubes, BD Biosciences)^73^. Prior to FACS sorting propidium iodide (PI) or Hoechst 33258 was added to a final concentration of 2 µg/mL to label the dead cells. Cells were analyzed and sorted on a FACS ARIA II (BD Biosciences). The data were analyzed with FlowJo data analysis software (Tree Star, Ashland, OR, USA).

### Immunoblotting

Glioblastoma xenografts proteins were extracted using cell lysis buffer (Cell Signaling) supplemented with proteinase and phosSTOP phosphatase inhibitor cocktail (Roche). Proteins were separated by SDS-PAGE and transferred onto polyvinylidene difluoride membranes, which were then probed with primary antibodies, followed by horseradish peroxidase-conjugated secondary antibody, and visualized by ECL (GE Healthcare). Antibodies specific for Hugl1 and tubulin as loading control were from Sigma.

### RNA extraction and reverse transcription

For all experiments, RNAs were extracted with QIAGEN RNeasy microkit and reverse transcribed into cDNAs using high capacity RNA to cDNA kit (Applied Biosystems).

### Taqman low-density arrays

For the Taqman low-density array experiment, 200–500 cells/population were sorted from the CC of young adult Ascl1-GFP and NG2-DsRed double-transgenic mice. TaqMan^®^ Array Micro Fluidic Card were designed for neural stem cell, oligodendrocyte progenitor cell and differentiated oligodendrocyte markers, as well as well selected ACD regulators. A PreAmp pool was designed for these specific primers and a preamplification reaction was set up to 14 cycles according to manufacturer’s instructions (Custom TaqMan^®^ PreAmp Pools Protocol, Applied Biosystems).

### Whole-genome microarrays

For the microarray experiments 200–500 Lgl1 knockout and wildtype OPC cells (PDGFRα and red fluorescent protein positive O4 negative), were extracted with the RNA plus micro kit (QIAGEN) and reverse transcription into cDNAs was performed using the High Capacity Reverse Transcription Kit (Thermofisher scientific). Sample quality was controlled using the Agilent Bioanalyzer and samples were loaded on GeneChipR Mouse Gene 2.0 ST Arrays (Affymetrix). Microarray data were analyzed using Partek^®^ Genomic Suite 6.6 with RMA normalization and differential gene expression detected with ANOVA. Ingenuity pathway analyses (QIAGEN bioinformatics) were used to identify targets and pathway differentially expressed between the *Lgl1* WT and *Lgl1* cKO samples.

### Real-time quantitative reverse transcription PCR

Receptor-mediated endocytosis regulator genes were validated by qRT-PCR on OPC cultured from *GFAPCre*, *dTom*^*fl/fl*^, *Lgl wt* (*n* = 6) vs. *Lgl1*^*fl/fl*^ (*n* = 6) mice using the following primers: Ap2a1 (5′–3′: GGAACCTCCTGGTGGATGTC, 3′–5′: AGGACCTGGGCTGAGGAAG); Epb41l2 (5′–3′: GCCCCAGTCAGGGAATTTCA, 3′–5′: TTACCGCTTACACCGGACAC); Hip1r (5′–3′: ATCTGGCCAGGAGCAGATTG, 3′–5′: AAGACTCGCACCTGTGTCTC) Eea1 (5′–3′: TCTTCAGAGGGTTTCATATGTCCC, 3′–5′: TGAGGCCTGTAGGTCTTGGA) Reps1 (5′–3′: GCAGATACTCCACCAACCAGT, 3′–5′: GTACTGTCGTCTGGTCCTGG); Syne1 (5′–3′: GACATGCAGGGAGTCACACA, 3′–5′: CTTTTTGCTGCTGGAGGCTG); Pld2 (5′–3′: ATCCTGAAGGCTCACGAACAG, 3′–5′: GAAGAAAGGGTGAAGGAGGCA); p19^Arf^ (Forward: 5′–3′: TGAGGCTAGAGAGGATCTTGAGAAG, Reverse: 5′–3′: GTGAACGTTGCCCATCATCATC); LLgl1 (exon 2–3 Forward: 5′–3′: CCACAAGACTGTGGAGCATGG, Reverse: 5′–3′: AAA-TGC-ATC-TGG-GTG-ACG-GT).

### Transferrin uptake

The CC of *GFAP-Cre*, *dTom*^*lox/lox*^, *Lgl1* ^*wt/wt*^(*n* = 4) vs. *Lgl1* ^*ox/lox*^ (*n* = 4) was extracted from young adult mice (P60). After enzymatic digestion with papain (10 min, 15 U/mL)^72^, cells were washed with cold wash solution (PBS containing 20 mM glucose and 1% BSA). Then Transferrin Alexa Fluor 488 conjugate (Molecular probes, T13342)^74^ was added at 25 µg/mL and incubated at 37 °C for 15 min. Non-internalized ligand was removed from the cell surface using three 5 min wash with ice-cold freshly prepared stripping buffer (PBS/0.15% BSA, pH 3.5 adjusted with HCL) and washed three times with cold wash solution. Data analysis to quantify changes in Transferrin fluorescence in dTom+ cells was performed using a FACS Aria II (BD Biosciences) and analyzed with FlowJo data analysis software (Tree Star, Ashland, OR, USA).

### Live staining of cells

Plated *Lgl1* cKO and WT OPC were incubated on ice at 4 °C for 2 h with α-NG2-EC (Rabbit, courtesy of Dr. Stallcup) in Neurobasal medium (3% BSA). After two washes with neurobasal medium, cells were incubated for 1 h with an Alexa488 goat α-Rabbit. For lysosome tracking, cultured OPC were incubated with CellLight Reagent BacMam 2.0 (Molecular probe) 24 h prior to live imaging targeting the early endosome Rab5a (C10586) and lysosome-specific protein Lamp1 (C10507) at a concentration of 50 PPC.

### Fluorescence time lapse microscopy

Time lapse movies were taken under a conventional widefield epifluorescence system (Nikon) equipped with a 37 °C incubation chamber and CO_2_ control for live cell imaging. Live-labeled cells were incubated in differentiation conditions and the system programed to take fluorescent pictures at the following frequencies: 1 frame/30 s to follow early NG2 endocytosis and 1 frame/1 h to follow its trafficking towards lysosomes.

### TIRF microscopy

Live labeled cells (NG2-EC) were plated in differentiation conditions and placed on a coupled incubator for live cell imaging (37 °C, 5% CO_2_) under the TIRF microscope. Fluorescent pictures were taken at different frequencies: 1 frame/30 min or 1 frame/30 s. TIRF was used to provide a mean to selectively excite fluorophore very near the solid surface (<100 nm) without exciting fluorescence from regions farther from the surface. Therefore, any Alexa488 positive dot in perfect focus was considered as a docking NG2 on the membrane region that is being imaged. Different quantities of NG2 recycling over time were represented as total corrected fluorescence measured using ImageJ.

### Recycling inhibition

*Lgl1* cKO OPC were treated with differentiation medium containing 10 μM Monensin (TOCRIS, 5223) for 4 h. Cells were monitored under TIRF for NG2 recycling monitoring during the first hour of treatment or were switched back to normal differentiation medium for 5 days before being fixed and immunostained for O4 and NG2. Endocytosis of the proteoglycan NG2 was followed using a mouse-anti-NG2.EC antibody against the extracellular domain of NG2 (courtesy of Dr. Stallcup). OPC cultures were incubated 24 h prior to live imaging with CellLight^®^ Reagents BacMam 2.0 (Molecular Probes) targeting the early endosome-specific protein Rab5a (ref C10586) of lysosome-specific protein Lamp1 (ref C10507) at a concentration of 50 PPC. Cells were starved 2 h prior to imaging in NBA medium, and 50 ng/mL of PDGF-AA was added right before imaging to induce endocytosis. Time-lapse movies were taken over time spans of 30–45 min with 30 s interval between the images to follow early endocytosis of NG2 antibody and 2–3 h with a 30 min interval to follow its internalization to the lysosome. After live imaging, cells were fixed 10 min in PFA4%, stained with the appropriate antibodies and imaged with a Nikon Ti Yokogawa CSU-22 spinning disk confocal equipped with a Plan Apo VC 60×/1.4 oil objective.

### Endocytosis inhibitor

Monensin (TOCRIS, 5223), a selective recycling endocytosis^75^, was used and was not toxic at a working concentration of 10 µM.

### Univariate Cox model

 The prognostic significance of high Lgl1 expression (≥median) on overall survival (defined as the time between diagnosis and death due to any cause) was retrospectively tested. The follow-up period ranged from 0 to 8 years (median = 1 year). The number of events that occurred during this period and the number of censored individuals are: 86 events, with 13 censored.

### Statistics and analysis

Statistical analyses were conducted as indicated in the figure legends using GraphPad Prism 5.0 and Microsoft Excel. A Mann and Whitney two-tailed paired *t*-test was conducted to analyze for significant differences between two treatment groups. For Kaplan–Meier survival rate analyses, the number of surviving mice were recorded daily. The data were subjected to Log-rank test in order to determine if significant differences existed in survival between the *Lgl1* knockout and control groups. For statistical tests *p < 0.05,**p < 0.01,***p < 0.001. A univariate Cox models was fitted on the survival data of the 99 glioma patients (92 GBM and 7 LGG) to obtain *p*-values. R-function ‘survfit’ (package ‘survival’) was used to generate survival curves from the previously fitted Cox model.

### Data availability

The data that support the findings of this study are available from the corresponding author upon reasonable request.

## Electronic supplementary material

Supplementary Information

Description of Additional Supplementary Files

Supplementary Movie 1
