# Supplementary material for: Lgl1 controls NG2 endocytic pathway to regulate oligodendrocyte differentiation and asymmetric cell division and gliomagenesis
Source: Nat Commun. 2018 Aug 21;9:2862. doi: 10.1038/s41467-018-05099-3 (PMC6104045; doi:10.1038/s41467-018-05099-3)
Supplement: Supplementary file 1 — Supplementary Information [file 41467_2018_5099_MOESM1_ESM.pdf]

Lgl1 controls endocytic pathway to promote oligodendrocyte progenitor differentiation and asymmetric division and suppresses gliomagenesis.

Daynac, Chouchane et al

Supplementary Figure 1

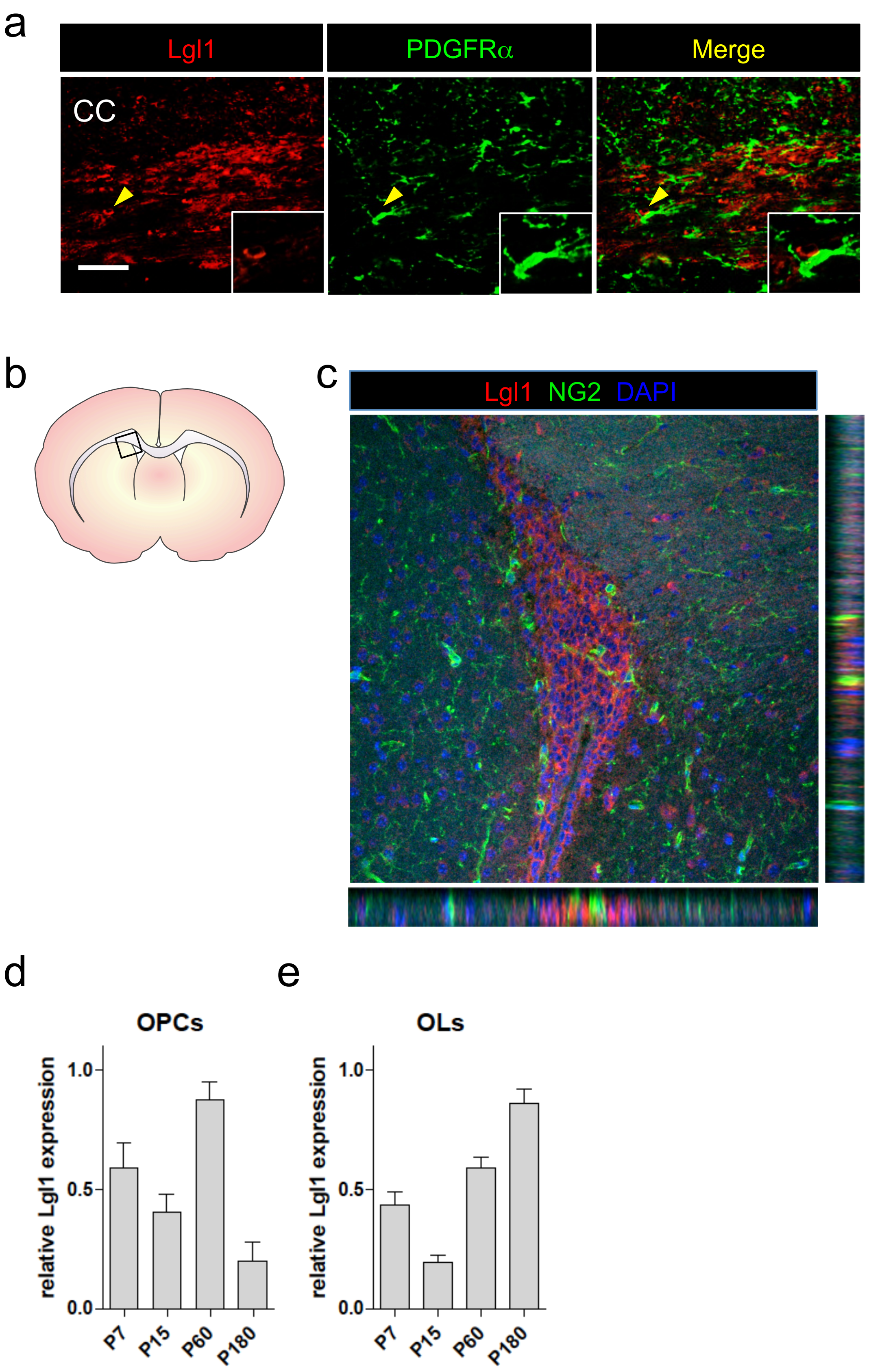

Supplementary Figure 1. Lgl1 is expressed in NG2-negative cells in the SVZ

(a) Co-immunostaining for Lgl1 and PDGFRα. The arrows point to an Lgl1-positive cell. Scale bar: 100 µm. (b) Drawing of a coronal section of adult mouse brain, the dorsal section of the SVZ is highlighted in the white square. (c) Representative confocal immunostaining of the dorsal section of the SVZ with Lgl1 (red) and OPC marker NG2 (green) showing low overlap between the two markers in the SVZ. (d) Graphs representing Lgl1 mRNA levels obtained by RT-qPCR at different ages in isolated corpus callosum OPC and (e) OL. n=4 animals per genotype. OPC: oligodendrocytes progenitor cells, OL: oligodendrocytes.

Supplementary Figure 2

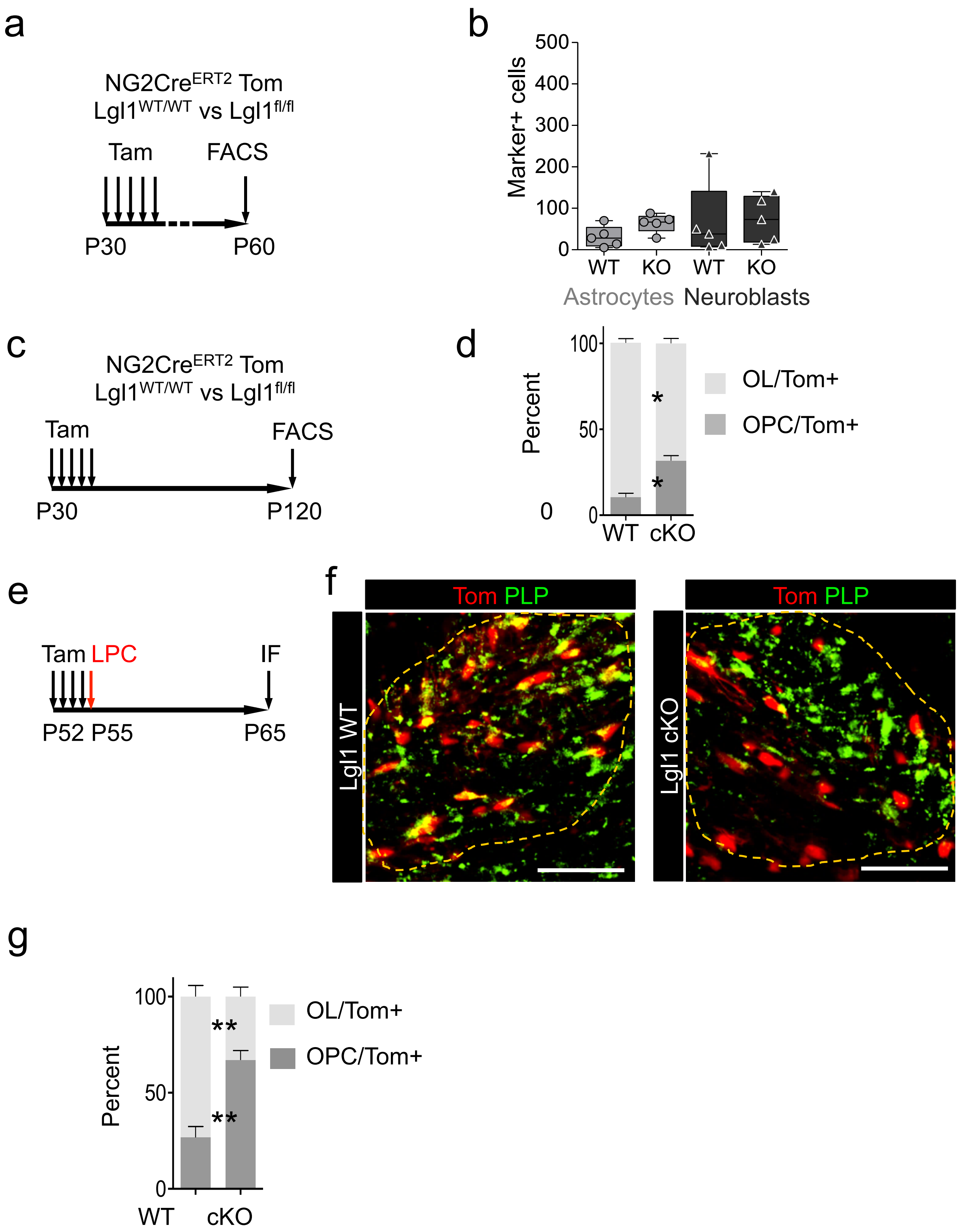

Supplementary Figure 2. *Lgl1* knockout OPC retain long-term immature fate without switching lineage potential.

(a) Experimental design for knocking out *Lgl1* in P30 mice, followed by FACS at P60 for astrocyte marker GLAST and neuroblast marker CD24. (b) Plot showing quantification of astrocytes (grey) and neuroblasts (dark grey) of *Lgl1* WT and cKO mice treated as shown in (a). Note that OPC are selectively increased in the *Lgl1* cKO mice (see Figs. 2 d, e), but astrocytes and neuroblasts are unchanged. (c) Schematic of broad deletion of Lgl1 in P30 mice followed by FACS for OL and OPC at P120. (d) FACS quantification depicting absolute numbers of OPC (grey) and OL (light grey) in the corpus callosum. *Lgl1* knockout (cKO) OPC show persistent reduced differentiation vs. *Lgl1* WT. Data are represented as mean +/- s.e.m from the analyses of 4 *Lgl1* WT vs 4 *Lgl1* cKO mice (Student´s t-test, *p<0.05). (e) Experimental design for knocking out *Lgl1* in demyelinated lesions induced by LPC injection. (f) Representative co-immunofluorescence for red fluorescent protein (RFP) to track OPC and progeny (Tom+) and mature OL marker Plp (green). Lesion is outlined by dashed line. Scale bar:100µm. (g) Graph representing the quantification of mature OL (Plp+) amongst all Tom+ cells in the lesion. Mature OL are decreased in the *Lgl1* cKO (73.3 +/- 5.7% OL in *Lgl1* WT vs. 33.0 +/- 4.9% in *Lgl1* cKO). Data are represented as mean +/- s.e.m from the analyses of 4 *Lgl1* WT vs. 4 *Lgl1* cKO mice (**p<0.01, Mann-Whitney test). Tam: tamoxifen, Tom: dTomato, OPC: oligodendrocytes progenitor cells, OL: oligodendrocytes, WT: wild type, cKO: conditional knockout, LPC: lysophosphatidylcholine.

.

Supplementary Figure 3

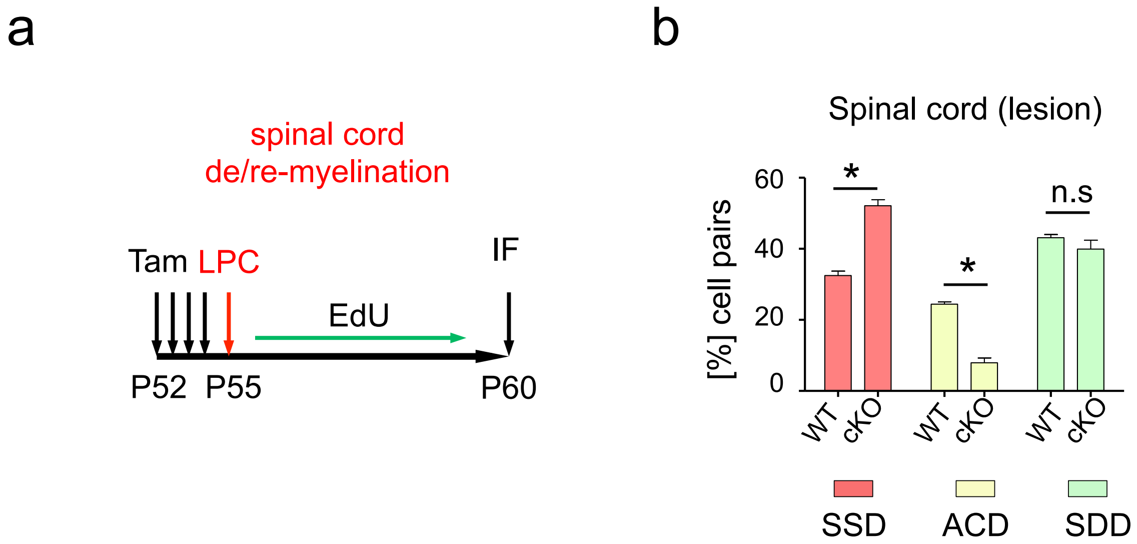

Supplementary Figure 3. Lgl1 loss increases OPC symmetric, self-renewing divisions at the expense of asymmetric divisions during spinal cord remyelination. (a) Experimental design of in vivo pair assays in demyelinated lesions in the spinal cord. (b) Percentage of symmetric and asymmetric divisions within lesion. Data are represented as mean +/- s.e.m from the analyses of 4 *Lgl1* Wt vs 4 *Lgl1* cKO mice (*p<0.05, Mann-Whitney test). LPC: lysophosphatidylcholine.

Supplementary Figure 4

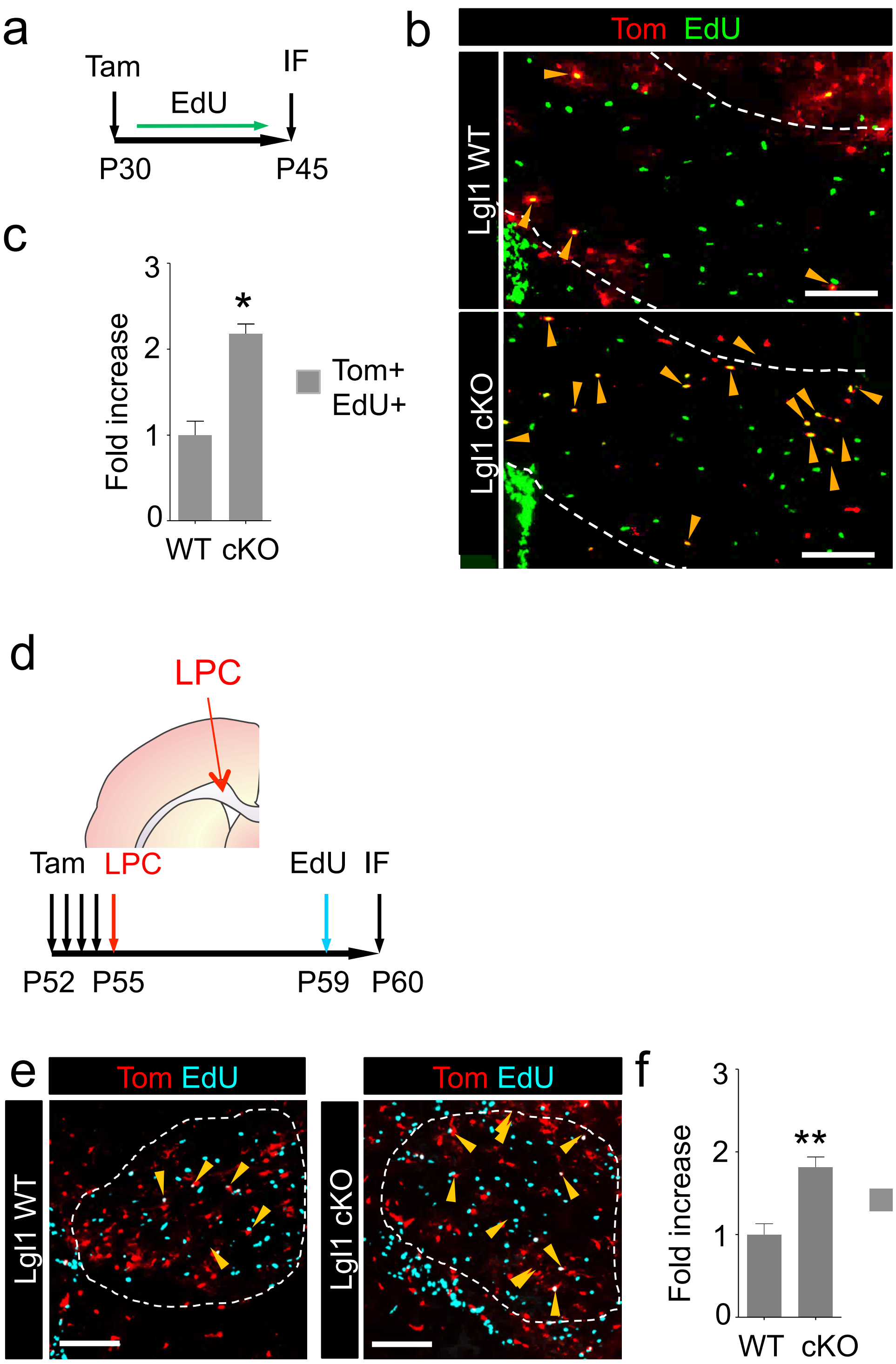

Supplementary Figure 4: Lgl1 inhibits OPC proliferation.

(a) Experimental design to induce clonal Lgl1 deletion and to quantify proliferation in NG2+ cells and progeny. P30 *NG2-Cre^{ERT2}*, *Tom Cre-reporter*, *Lgl1$^{fl/fl}$* and *NG2-Cre^{ERT2}*, *Tom Cre-reporter, Lgl1$^{wt/wt}$* triple transgenic mice were injected with tamoxifen once, subsequently treated with EdU in drinking water for 14 days and brains were subjected to immunofluorescence (IF). (b) Immunofluorescence for red fluorescent protein to label and track NG2+ and progeny (Tom+) cells and cells positive for proliferation marker EdU. Sections were from *Lgl1* WT and *Lgl1* cKO mice, treated as described in (a). Orange arrowheads point at Tom+ EdU+ cells (EdU and Tom). Scale bars: 100µm. (c) Graph showing the fold-increase of EdU+ Tom+ cells in the corpus callosum of *Lgl*1 cKO mice compared with *Lgl1* WT mice. Data are represented as mean +/- s.e.m of 4 Lgl1 WT vs. 4 Lgl1 cKO mice (*p<0.05, Mann-Whitney test). (d) Experimental approach to delete Lgl1 in NG2+ cells, and induce lesion in the spinal cord by injecting LPC and assess proliferation. (e) Immunofluorescence of a section from a lesioned spinal cord for red fluorescent protein (Tom), EdU and DAPI, where the lesioned area is outlined with a white line. Scale bars: 200µm. (f) Graph showing a significant increase of Tom+ EdU + cells in *Lgl1* cKO compared with *Lgl1* WT spinal cord lesions. Data are represented as mean +/- s.e.m of 3 *Lgl1* WT vs 4 *Lgl1* cKO mice (*p<0.05, Mann-Whitney test). Tom: Tomato, LPC: lysophosphatidylcholine.

Supplementary Figure 5

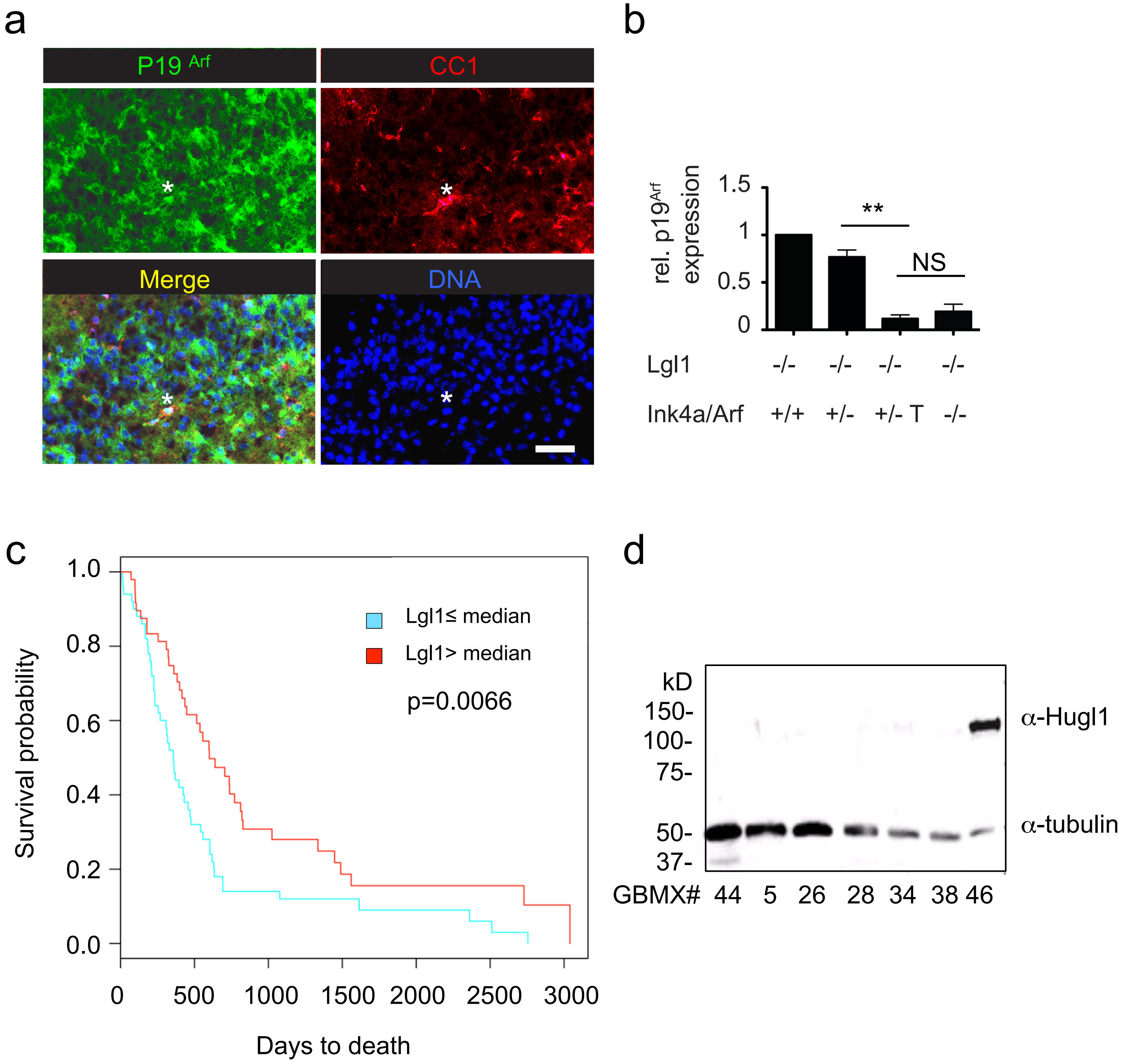

Supplementary Figure 5. p19^{Arf} is expressed in normal brain and Lgl1 expression is lost in GBM. (a) Co-immunofluorescence staining for p19^{Arf} and CC1. Asterisk marks p19^{Arf} and CC1 double-positive cell in the cortex of postnatal day 30 mouse. Five consecutive sections from two P30 mice each were analyzed, showing the same results. Scale bar = 50 μM. (b) Relative p19^{Arf} expression in Lgl1 KO (-/-) Ink4a/Arf wildtype (+/+), heterozygous (+/-) and homozygous (-/-) OPC and tumor cells (T), derived from Lgl1 KO Ink4a/Arf +/- mice (corresponding to Fig. 4), determined by RT-qPCR. Assays were done in triplicates from one cell line for each genotype. (**$p<0.01$, Mann-Whitney test; NS=not significant). (c) Competing survival curve for patients with high (red) and low (green) expression of the human Lgl1 homolog Hugl1 (hazard ratio0 0.60, $p=0.0066$, Univariate Cox model). (d) Immunoblotting for Hugl1 showing that the protein is lost in the majority of human GBM xenografts. Anti-tubulin was detected as loading control. GBM=glioblastoma multiforme.

Supplementary Figure 6

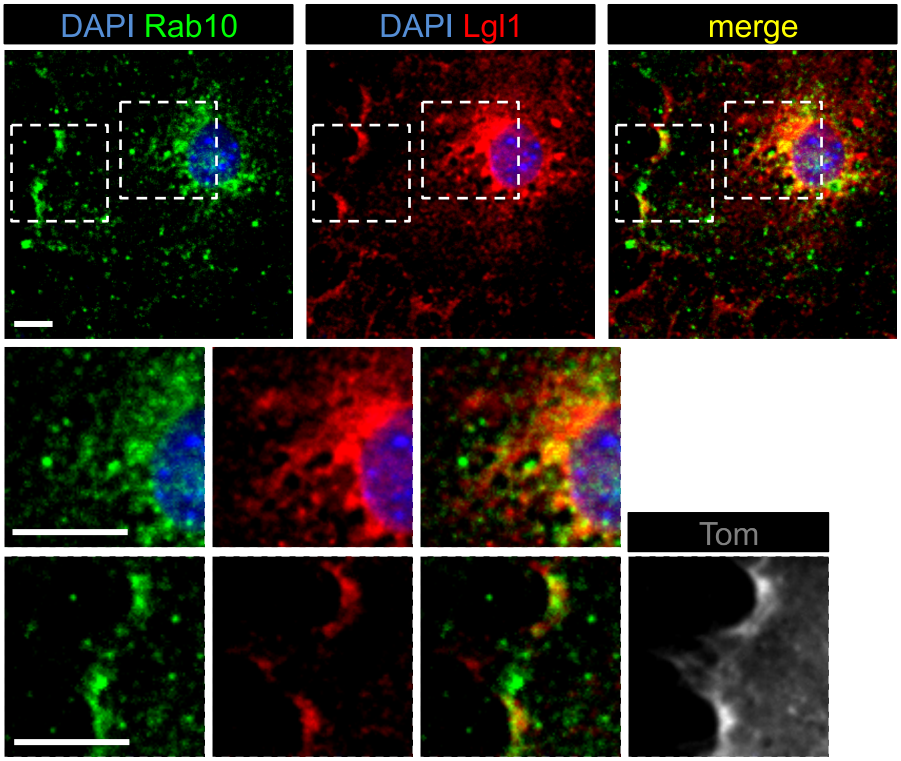

Supplementary Figure 6: Lgl1 colocalizes with early endosome marker Rab10 both at the membrane and perinuclear.

Immunostainings of Lgl1 (red) and early endosome marker Rab10 (green) showing coexpression both at the membrane and around the nucleus in *Lgl1* WT OL. Higher magnifications are presented in dashed squares.

Supplementary Figure 7

a

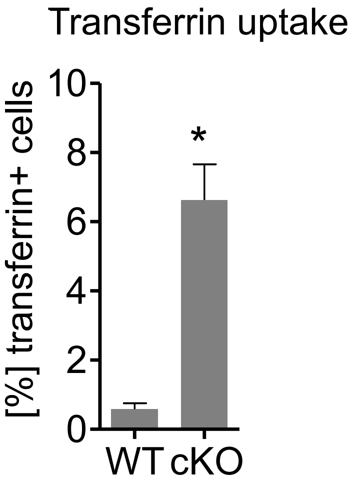

Supplementary Figure 7: Higher transferrin uptake in Lgl1 cKO OPC.

Graph representing the frequency of transferrin uptake in acutely isolated OPC by FACS. *Lgl1* knockout OPC (cKO) show significantly increased endocytosis of transferring. (Student´s t-test, *P<0.05). Data represented as mean +/- s.e.m from 5 *Lgl1* WT vs. 5 *Lgl1* cKO mice.

Supplementary Table 1

	Corpus Callosum (no lesion)		Corpus Callosum (lesion)		Spinal Cord (lesion)	
	WT	cKO	WT	cKO	WT	cKO
SSD	28.4 ± 8.3%	**51.2 ± 5.0%**	37.9 ± 3.1%	**50.8 ± 6.0%**	32.5 ± 2.2%	**52.1 ± 2.9%**
ACD	23.2 ± 6.3%	**12.5 ± 3.4%**	28.5 ± 4.9%	**9.8 ± 3.8%**	24.4 ± 1.1%	**7.9 ± 2.3%**
SDD	48.4 ± 9.4%	36.3 ± 8.2%	33.6 ± 5.9%	39.4 ± 9.2%	43.1 ± 1.6%	39.9 ± 4.3%

Supplementary Table 1. Frequency of cell division modes in the intact and remyelinating brain and spinal cord. Cells division mode types in the intact corpus callosum (corresponding to Figure 3c), the lesioned, remyelinating corpus callosum (corresponding to Figure 3d) and the lesioned, remyelinating spinal cord (corresponding to supplementary Figure 3b). In bold are significant changes in cell division modes observed in *Lgl1* cKO mice vs. *Lgl1* WT control mice. SSD=symmetric, self-renewing divisions; ACD=asymmetric cell divisions; SDD=symmetric, differentiating divisions
