# Supplementary material for: Lgl1 controls NG2 endocytic pathway to regulate oligodendrocyte differentiation and asymmetric cell division and gliomagenesis
Source: Nat Commun. 2018 Aug 21;9:2862. doi: 10.1038/s41467-018-05099-3 (PMC6104045; doi:10.1038/s41467-018-05099-3)
Supplement: Supplementary file 2 — Description of Additional Supplementary Files [file 41467_2018_5099_MOESM2_ESM.pdf]

Description of Additional Supplementary Files

File Name: **Supplementary Movie 1**
Description: **Continuous endocytosis and recycling of NG2 in Lgl1 cKO cells**
TIRF video microscopy of Lgl1 cKO cells performed at constant conditions of 37°C and 5% CO2. Cells are live-labeled for NG2-EC primary antibody revealed by a secondary antibody conjugated to Alexa Fluor 488. Following incubation of cells in differentiation medium, fluorescent images are acquired every 20 minutes up to 10 hours. NG2 is visualized as white dots. Note the changes in both fluorescence and focus of these dots threw the time line suggesting a continuous endocytosis and recycling of NG2 in Lgl1 cKO cells.
